# An Excitation Strategy for the Initial Condition Generation for Surface Hopping Trajectories Using Electron-Only Dynamics Including Explicit Laser Pulses

**DOI:** 10.1021/acs.jctc.5c01390

**Published:** 2025-12-08

**Authors:** Lorenz Grünewald, Laurens van Dam, Sebastian Mai

**Affiliations:** † Institute of Theoretical Chemistry, Faculty of Chemistry, University of Vienna, 1090 Vienna, Austria; ‡ Vienna Doctoral School in Chemistry (DoSChem), Faculty of Chemistry, University of Vienna, 1090 Vienna, Austria

## Abstract

Nonadiabatic dynamics simulations, e.g., via trajectory surface hopping, are nowadays used regularly to describe various photoinduced phenomena in molecules. For a number of reasons, in the setup of such simulations, the actual photoexcitation process is often described by rather crude and approximate excitation schemes (e.g., vertical excitation by an implicit delta pulse), and more attention is directed to the ensuing dynamics simulations. However, several studies have implied the importance of properly considering the spatial, temporal, and spectral details of the exciting laser pulse. Here, we suggest the “electron-only explicit” (“EOE”) excitation scheme for setting up trajectory surface hopping initial conditions based on an explicit laser pulse at little computational cost. The scheme is based on solving the time-dependent electronic Schrödinger equation including the explicit influence of a laser pulse within the frozen-nuclei approximation. The obtained time-dependent, coherently excited, electronic populations are then used to stochastically select the initial electronic states in a postprocessing step. Here, the electronic populations are renormalized such that one excites a reasonable fraction of the initial condition even when operating well within the weak-field regime. The new scheme is made freely available as part of the SHARC 4 dynamics package. We illustrate and validate the new excitation scheme by means of several simulations of sodium iodide and 6-cyanobenzquinuclidine excited with laser pulses of different energy and pulse duration, comparing to quantum dynamics results.

## Graphical abstract

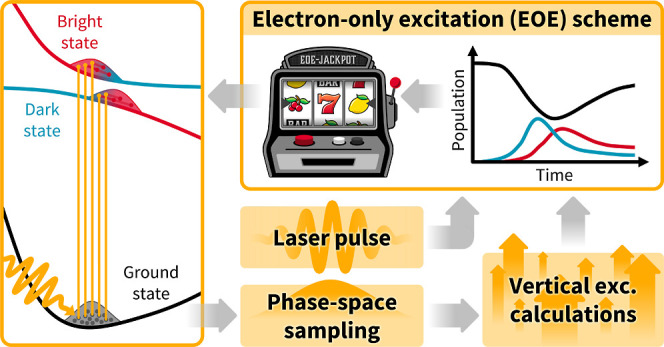

## 1 Introduction

The development of ultrafast laser spectroscopy has enabled tracking—and potentially steering—molecular dynamics on (sub)-femtosecond time scales.^1–6^ Still, the interpretation of the molecular dynamics during and after photoexcitation is highly nontrivial and challenging due to the intrinsic interaction of all nuclear and electronic degrees of freedom.

Nonadiabatic molecular dynamics (NAMD) simulations that specifically incorporate the photoexcitation process and the subsequent processes can significantly contribute in understanding ultrafast processes. One prominent example for such an NAMD method is the trajectory surface hopping (TSH) method.^7–10^ Within this mixed quantum-classical method, a swarm of trajectories is propagated, in which nuclei follow classical trajectories driven by the gradient of a single adiabatic state that can stochastically switch (hop) based on the quantum mechanical evolution of the electronic wave function. To explicitly account for the photoexcitation process in TSH, in principle, one could start the trajectories in the ground state and incorporate the interaction between the molecule and a laser field in the dynamics^11–16^ (we will refer to this as the “full explicit” excitation scheme below). However, this is rarely done because a considerable part of the computational effort would be wasted on propagating trajectories in the ground state that never get excited by realistically weak laser pulses.^11–22^ Enhancing the number of excited trajectories and thus the dynamics efficiency by artificially increasing the laser intensity is reported to lead to spurious behavior because of strong-field and interference effects.^14,16^ Moreover, especially for longer laser pulses, interference effects between the ground-state and excited wave functions break the underlying assumptions of TSH, leading to inaccurate results and making the “full explicit” scheme undesirable.^14,23^

Instead of explicitly treating the light-matter interaction in the TSH simulations, one can approximate the photoexcitation process in a cheaper way by starting the trajectories in suitably selected initial excited states.^7,24–26^ In most TSH simulations, this is done by invoking a sudden vertical excitation,^26–28^ which we refer to as “vertical” excitation scheme in the following. It treats photoexcitation as an instantaneous event and thereby neglects the true spectral and temporal structure of the exciting laser pulse.^29^ Although such “vertical” excitation schemes often consider an energy window around the central pulse frequency to restrict the set of potential initial states, there is no standard criterion for the window extent and the allowed excitation region is—to some extent—arbitrarily chosen, e.g., via the window bandwidth.^29,30^ While such a relatively crude treatment of photoexcitation may suffice in certain contexts and may describe photochemical processes reasonably well—e.g., if the focus of the simulation is on photodynamics that happens in a statistical manner hundreds of femtoseconds after an excitation pulse—it potentially fails for scenarios where the dynamics on the time scale of the laser pulse is relevant.

Recently, Janoš et al.^24^ highlighted the importance of carefully selecting initial conditions for trajectory-based NAMD simulations and emphasized how the commonly used “vertical” excitation scheme can produce misleading results that do not take into account the laser properties. Hence, they suggested the promoted density approach (called the “PDA” scheme below) to incorporate the temporal and spectral profile of a laser pulse in the selection of the initially excited states.^29^ The “PDA” is theoretically well founded and computationally inexpensive, and can describe a wide variety of photoexcitation scenarios, including excitation by chirped laser pulses. However, there are some aspects that this method does not fully describe. For example, the available implementation^31^ does not currently consider the (static or time-dependent) polarization of the laser, although it could in principle be included in the algorithm.^29^ Moreover, the “PDA” implementation implicitly assumes the electric dipole approximation, which truncates the multipole expansion of the light-matter interaction after the first term, and a plane-wave description of light. Including higher-order terms can be of interest when considering short wavelengths, structured light, tightly focused laser beams, or other nonplane-wave scenarios.^32–34^ Lastly, the “PDA” does not consider the simultaneous/competing electronic dynamics that might occur during the pulse duration due to other types of couplings besides light-matter interactions, for example, strong spin–orbit couplings that lead to few-fs intersystem crossing.^35^ However, the mentioned physical phenomena—polarization, structured light, and intersystem crossing—can be critical for the description of the photoinduced dynamics, e.g., in transition metal or lanthanide complexes.^34,36^

In this work, we propose a complementary excitation scheme, the “electron-only explicit” (“EOE”) scheme, for preparing initial conditions for TSH simulations. The design goals of this scheme are (i) to include the effect of the exciting laser at the level of the initial conditions, (ii) to require only one single-point calculation per initial condition to have similar computational cost as other excitation schemes, (iii) to include laser fields with arbitrary polarization and potentially beyond the dipole approximation, and (iv) to consider that other couplings besides light–matter interactions could be active during the excitation. Our method is based on two steps, where first the photoexcitation is simulated explicitly by propagating the electronic wave function in the presence of a laser field for each initial condition, assuming frozen nuclei. In a second step, based on the evolution of the electronic wave function coefficients, the initial electronic states and starting times^29^ for each initial condition are selected stochastically, where the coefficients are renormalized to increase the number of accepted initial conditions. This renormalization solves the problem that otherwise weak laser fields would yield only very few excited initial conditions.^16^ Subsequently, the selected initial conditions are propagated with regular TSH, just like after using other excitation schemes. Our method is implemented in the SHARC 4 package^37^ and offers high computational efficiency due to the use of frozen nuclear coordinates during the laser interaction simulations. At the same time, it naturally incorporates laser beam polarization and is compatible with focused beams and nonplane-wave propagation regimes,^38–41^ e.g., in scenarios where a nearly isolated magnetic field occurs.^32–34^ While we discuss the “EOE” scheme in the context of TSH, we note that our proposed photoexcitation scheme can also be coupled to the coherent switching with decay of mixing (CSDM) approach,^42^ combining advantages of the Ehrenfest method and TSH. We demonstrate the validity and applicability of our method by analyzing the photodynamics of sodium iodide (NaI) and 6-cyanobenzquinuclidine (CBQ) excited by a variety of Gaussian laser pulses.

## 2 Theory

In this section, we start by briefly recapitulating the general framework of TSH and provide relevant equations. We then proceed to summarize the currently used photoexcitation schemes in TSH to provide context for the proposed “EOE” excitation scheme. Finally, we derive the key equations and parameters for the new excitation scheme.

### 2.1 Surface Hopping

The TSH approach denotes an NAMD method that treats the intertwined electronic and nuclear dynamics within a mixed quantum-classical picture.^7^ Due to its conceptual simplicity, efficiency, and accuracy, TSH is nowadays among the most widely used flavors to perform NAMD.^9^

The method considers the nuclear motion to follow classical Newtonian mechanics, while the electronic dynamics is described by a time-dependent electronic Schrödinger equation. The involved time-dependent electronic wave function is constructed as a linear combination of the eigenfunctions of the electronic Hamiltonian for the current nuclei configuration *R⃗*

$$|\Psi \left[\mathrm{R⃗}\left(t\right),t\right]\rangle =\underset{\alpha }{\sum }c_{\alpha }\left(t\right)|\phi_{\alpha }\left[\mathrm{R⃗}\left(t\right)\right]\rangle ,$$ 1

in which α runs over all basis states in the set of included states, *c*_α_(*t*) are time-dependent complex coefficients, and $|\phi_{\alpha }\left[\mathrm{R⃗}\left(t\right)\right]\rangle$ are the electronic basis states. At this point, one is free to choose a basis for the wave function, e.g., a (quasi-) diabatic basis that is constant at all positions or the adiabatic basis consisting of the eigenfunctions of the electronic Hamiltonian *Ĥ*.^43^ We will comment below on the implications of this freedom of choosing the basis representation for the new excitation scheme.

The wave packet expansion can be inserted into the time-dependent Schrödinger equation to obtain an expression for the electronic equation of motion

2 $$\frac{\partial c_{\beta }\left(t\right)}{\partial t}=-\sum_{\alpha }\left[iH_{\beta \alpha }+K_{\beta \alpha }\right]c_{\alpha }\left(t\right),$$

where β is one electronic state, $H_{\beta \alpha }=\left\langle \phi_{\beta }\left|Ĥ\right|\phi_{\alpha }\right\rangle$ is a matrix element of the electronic Hamiltonian operator over two basis states, and $K_{\beta \alpha }=\left\langle \phi_{\beta }\left|\frac{\partial }{\partial t}\right|\phi_{\alpha }\right\rangle$ is an element of the time-derivative coupling matrix.^9,44^ Here, different choices for *Ĥ* are possible; the simplest includes only the electron kinetic energy and the intermolecular Coulomb interactions (the molecular Coulomb Hamiltonian, MCH).^43^ However, one can also include, e.g., spin–orbit couplings or light-matter interactions (which would make *Ĥ* time-dependent).

The actual TSH simulation is based on an iterative algorithm, involving several steps. First, the nuclear positions *R⃗* are updated based on the electronic forces. These forces are computed as gradients of the current “active state” β, which is one of the electronic basis states. The active state is determined stochastically from the evolution of the electronic coefficients *c*_α_ of all states α. A change of active state is referred to as a “surface hop”.

At the new nuclear positions, an electronic structure calculation is performed, the obtained gradients are used to update the velocities *v⃗*, and the energies and couplings are used to propagate the electronic coefficients using eq 2. Based on the fewest-switches surface hopping concept,^7^ several prescriptions to compute the hopping probabilities have been proposed.^7,43,45,46^ In this work, we adopt the global flux surface hopping (GFSH) scheme,^46^ which requires only the knowledge of the coefficients of the previous and current time steps, but not of any other intermediate. The GFSH hopping probability *P*_β→α_ is computed as

3 $$P_{\beta \to \alpha }\left(t+\Delta t\right)=\left(1-\frac{{\left|c_{\beta }\left(t+\Delta t\right)\right|}^{2}}{{\left|c_{\beta }\left(t\right)\right|}^{2}}\right)\times \frac{{\left|c_{\alpha }\left(t+\Delta t\right)\right|}^{2}-{\left|c_{\alpha }\left(t\right)\right|}^{2}}{\sum_{\gamma }\;\max\left[0,{\left|c_{\gamma }\left(t+\Delta t\right)\right|}^{2}-{\left|c_{\gamma }\left(t\right)\right|}^{2}\right]},$$

where the sum in the denominator effectively goes only over all states γ whose population increases from *t* to *t* + Δ*t*.^46^ The active state-active state probability *P*_β→β_ as well as all negative probabilities *P*_β→α_ < 0 are set to zero. In eq 3 the first factor on the right-hand side is the overall probability to leave β according to the fewest-switches criterion,^7^ and the second factor splits this probability over the possible new states α.^46^

In each time step, after the hopping probabilities were computed from the coefficients, the new active state is stochastically selected by drawing a random number *r*(*t*) between 0 and 1.^47,48^ A hop to a prospective new active state α is performed if

4 $$\sum_{\gamma =1}^{\alpha -1}P_{\beta \to \gamma }\left(t\right)<r\left(t\right)\leq P_{\beta \to \alpha }\left(t\right)+\sum_{\gamma =1}^{\alpha -1}P_{\beta \to \gamma }(t).$$

Although not relevant for the proposed method below, after the active state selection, in most TSH simulations further steps are performed (e.g., momentum rescaling, decoherence correction).^10,49^ Lastly, the nuclear forces are updated for the new active state, and the next time step is entered. This procedure generates a trajectory, i.e., a time series of nuclear positions *R⃗*(*t*), velocities *v⃗*(*t*), electronic populations |*c*_α_(*t*)|^2^ for all states α, and other time-dependent quantities.

Because TSH is a stochastic, classical approach, one needs to simulate a swarm of trajectories. The starting point for these trajectories are defined by the so-called initial conditions, which consist of a distribution of initial coordinates ${\mathrm{R⃗}}_{k}\left(0\right)$ , initial velocities ${\mathrm{v⃗}}_{k}\left(0\right)$ , and an initial electronic state β_*k*_(0) for each initial condition *k*. These initial conditions describe the molecular system at time zero, e.g., its equilibrium distribution at some experimental conditions. The initial position–momentum pairs ${\left(\mathrm{R⃗}\left(0\right),\mathrm{v⃗}\left(0\right)\right)}_{k}$ are usually sampled via adiabatic MD simulations,^50,51^ from a Wigner distribution of an approximate nuclear wave function,^51–54^ or, e.g., more elaborated, from a quantum-thermostatted ab initio MD simulation, as suggested by Janoš et al.^24^

To complete the set of initial conditions, one needs to select an initial electronic state β_*k*_(0) for each trajectory *k*. Typically, this step is made such that the simulations resemble some experiment in which the molecule is photoexcited in a particular way. There are various excitation schemes in use, as already mentioned briefly above, to select the initial active state. Figure 1 provides an overview about the schemes that we discuss here. All schemes involve a ground state sampling, an initial state selection, and the actual dynamics simulation as the bare minimum. Some approaches involve additional steps, as described below.

The first approach, which we label “hand picking” here, involves the user manually choosing an excited state for all trajectories. This is a trivial (Figure 1a) but not very realistic option in most situations and is only mentioned as the simplest option.

One of the most common excitation schemes,^26,30^ the “vertical” excitation scheme, simulates implicitly the action of an infinitely short pulse that instantaneously excites the molecule. In practice, this scheme uses the excitation energies and oscillator strengths of all states of all initial conditions (obtained from excited-state calculations), i.e., the set $\left\{{\left(\Delta E_{\alpha },f_{osc,\alpha }\right)}_{k}\right\}$ , to compute selection probabilities for all states (Figure 1b). Most often, the probabilities are computed as $\frac{f_{osc,\alpha }}{\Delta E_{\alpha }^{2}}$ (assuming constant intensity at all energies^55^) or $\frac{f_{osc,\alpha }}{\Delta E_{\alpha }}$ (assuming constant photon flux at all energies^55^), and are then normalized to the highest probability. If an energy window is used, only transitions within the window are considered for computing the probabilities and the normalization. For each state α of each initial condition *k* it is then stochastically decided whether this state is used as an initial active state. This approach is popular due to its simplicity and computational efficiency—for each initial condition *k*, only a single-point vertical excitation calculation needs to be carried out. However, the approach does not represent the exciting laser pulse very well. For example, due to the use of (isotropically averaged) oscillator strengths, the scheme does not consider any polarization effects. Often, the set of considered states is restricted to states whose excitation energy falls into a certain energy window,^26^ but this is a crude approximation of the spectrum of a pulse and is also inconsistent with the assumption of an infinitely short pulse. Moreover, the “vertical” excitation scheme cannot describe pulses with specific temporal, spectral, or temporal-spectral distributions.

The “PDA” scheme^29^ (Figure 1c) significantly improves upon the “vertical” excitation scheme by explicitly considering the time–energy (Wigner) distribution^56^ of an exciting laser pulse. The core idea is to use the Wigner distribution value at a randomly sampled excitation time *t*′ and at the excitation energy of the considered state as a factor in the selection probabilities of the “vertical” excitation scheme. In this way, it is more likely to select excited states near the maxima of the pulse’s spectrum and temporal envelope. One advantage of this approach is that it generally also works for chirped pulses. The “PDA” scheme requires the same electronic structure data as the “vertical” excitation scheme and additionally requires the definition of a laser pulse as well as the computation of its Wigner distribution. As the latter is typically inexpensive, this scheme has essentially the same computational cost as the “vertical excitation” scheme but provides a more realistic description of the excitation process.

**Figure 1:**
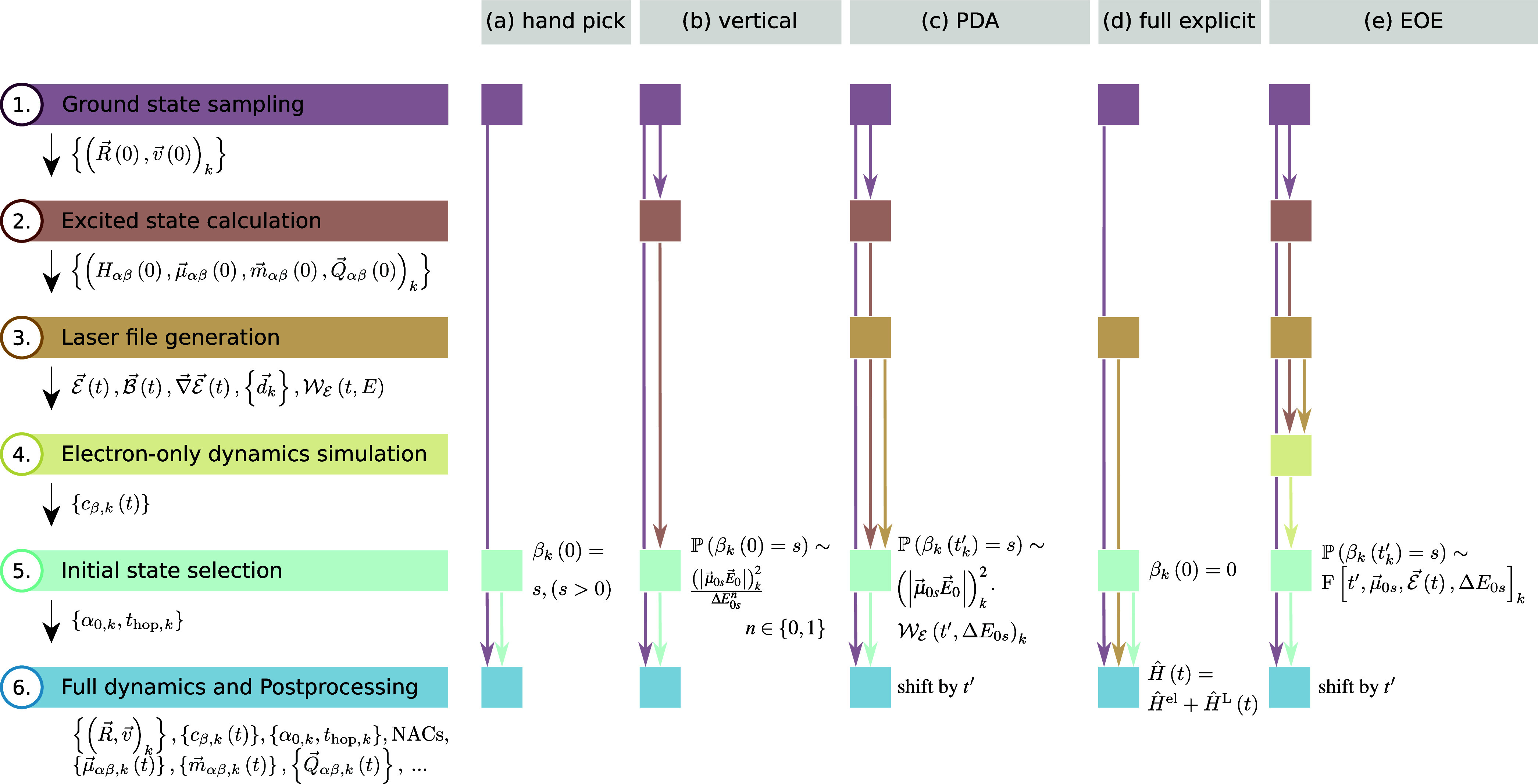
Overview over the algorithmic steps in various different excitation schemes, as described in the text: (a) “hand picking”, (b) “vertical”, (c) “PDA”, (d) “full explicit”, and (e) “EOE”. The left-most column labels the different steps and indicates the quantities that are computed in that step. The other columns indicate which steps are part of the given algorithm. The main step—the initial state selection—is labeled with the key equation for how the initial states are determined.

An alternative to the implicit excitation schemes (i.e., the “hand picking”, “vertical”, and “PDA” schemes) is to include the laser pulse directly and explicitly in TSH simulations that are initiated in the ground state. In this “full explicit” scheme (Figure 1d), the electronic propagation needs to include an additional Hamiltonian term

5 $$Ĥ={Ĥ}^{MCH}+{Ĥ}^{L}(t),$$

$${Ĥ}^{L}\left(t\right)=(-\overset{n_{el}}{\underset{i}{\sum }}{\mathrm{r⃗}}_{i}+\overset{n_{nuc}}{\underset{A}{\sum }}Z_{A}{\mathrm{R⃗}}_{A})\;\cdot \;\overset{\to }{E}\left(t\right),$$ 6

in which ${Ĥ}^{L}$ is the light–matter interaction Hamiltonian, which is here given exemplarily as the electric dipole interaction operator. Instead, more complicated expansions of the light–matter interaction Hamiltonian could be included. With the expanded Hamiltonian, the TSH simulation can undergo field-induced hops that can excite trajectories naturally from the ground state without relying on any approximated excitation scheme. In general, this scheme can incorporate arbitrary electronic Hamiltonians that describe a broad variety of physical processes, making it formally very flexible, although it is limited by the neglect of interference effects, as mentioned in the introduction.^14,23^ Unfortunately, in typical weak-field regimes, the probability for field-induced hops is very low (often below one percent^29^), so the vast majority of the simulated trajectories will not reach the excited state and thus not contribute to the nonadiabatic dynamics simulation. Considering the cost of (ab initio) TSH simulations, this is very wasteful. However, it is not possible to identify those trajectories that would hop into the excited state a priori.

### 2.2 Electron-Only Explicit Excitation Scheme

The main goal of the “EOE” excitation scheme, which we propose here, is to provide a compromise between the inefficient but flexible “full explicit” scheme on the one hand and the efficient but more limited implicit schemes (“vertical” excitation and “PDA”) on the other hand. Like the “full explicit” scheme, the new scheme also explicitly propagates the electronic wave function in the presence of a laser pulse, but in a first step in a short and cheap dynamics simulation. From the results of this simulation, the initial active state is selected and the actual, field-free dynamics simulations are set up in a second step. This makes the “EOE” scheme essentially an approximation to the “full explicit” scheme, with the same theoretical background and justification as well as the same limitations.

Efficiency is increased in two ways to match approximately the effort needed for the implicit methods. First, the propagation including the laser pulse is carried out with frozen nuclear positions, which is a reasonable approximation for sufficiently short pulses and considering that the ground state nuclear density is time-independent in the absence of the laser pulse.^16,29^ With the nuclei frozen, the new scheme requires essentially the same data as the implicit schemes, i.e., electronic structure data (excitation energies and transition dipole moments) from one single-point calculation per initial condition. Because of this approximation, we chose to label the new scheme the “electron-only explicit” excitation scheme. The second measure to increase efficiency is that, after the frozen-nuclei/electron-only propagation is finished, we renormalize the time-dependent electronic populations of all electron-only simulations to effectively increase the amount of transferred population and thus the acceptance ratio of the initial conditions. We then apply surface hopping to the time-dependent, renormalized populations to stochastically select the initial active state α_*k*_(0) as well as a starting time *t*_*k*_^′^. Here, we use the GFSH prescription^46^ for the hopping probabilities because it only requires the electronic populations and thus can be applied a posteriori. As detailed below, this renormalization scheme allows to stay within the weak-field regime and nonetheless excite a considerable fraction of the initial conditions.

The “EOE” excitation scheme has been implemented in the SHARC 4 package^37^ in two new Python scripts. The first of the two scripts interactively leads the user through the setup of the electron-only dynamics simulations, reading the initial conditions, excited-state calculation results, and the laser file (see Figure 1e). After the electron-only simulations are finished, the second script samples the initial states and starting times, as described below, and produces an initial condition file from which the TSH simulations can be set up as usual within SHARC. The two new Python scripts—together with several smaller changes in the package to enable trajectory analysis including shifted starting times—are available under the GNU public license as part of the SHARC 4.0.2 release;^57^ full documentation will be available in the upcoming SHARC 4.1 major release. A brief workflow for setting up a dynamics simulation with the new “‘EOE” excitation scheme is given in Section S1 in the Supporting Information.

#### 2.2.1 Preparation Steps

The workflow scheme of our explicit laser excitation protocol is depicted in Figure 1e. The first three steps are identical to the ones necessary for the “PDA” scheme. First, as with other excitation schemes, the ground state sampling is carried out with any suitable method.

Second, during the excited-state single-point calculations, (excitation) energies and (transition) dipole moments should be computed. In our scheme, one can in principle also include off-diagonal couplings *H*_βα_ in the Hamiltonian, e.g., spin–orbit couplings. Furthermore, we are currently expanding SHARC to also consider magnetic (transition) dipole moments ${\mathrm{m⃗}}_{\beta \alpha }$ and electric (transition) quadrupole moments ${\mathrm{Q⃡}}_{\beta \alpha }$ in the light-matter Hamiltonian.

Third, one needs to specify a laser pulse, similar to what is needed for the “PDA” and “full explicit” schemes. Here, a numerical representation is used, where the corresponding laser file contains the electric field $\overset{\to }{E}\left(t\right)$ of the pulse for all time steps. It is not required to have an analytical closed-form equation for the electric field. With the expanded light-matter Hamiltonian, one would need to obtain not only the electric field in time, but also the magnetic field $\overset{\to }{B}\left(t\right)$ and electric field gradient $\overset{\to }{\nabla }\overset{\to }{E}\left(t\right)$ . While the time-dependent electric field can conveniently be computed from simply analytical formulas (e.g., carrier times envelope, as done below), consistent magnetic fields and electric field gradients are most easily obtained from finite-difference time-domain simulations of the laser pulse in three dimensions.^33,58–60^ In our scheme, we can optionally also generate random polarization directions ${\mathrm{d⃗}}_{k}$ for each initial condition *k* to simulate the effect of random orientation of the molecule. It is not required to compute the Wigner distribution of the pulse, which has the benefit that the “EOE” scheme is unaffected by negative regions of the Wigner distribution of some pulses.^56,61^

As we describe in the next subsection, the “EOE” scheme renormalizes the electronic populations. Hence, the precise field strength of the laser field does not significantly influence the selection probabilities. Therefore, the scheme can be used with very weak fields, for which strong-field effects can effectively be excluded.^14,16^ We expect that laser fields exciting much less than one percent of the population should work well (however, the required field strength might still depend significantly on the molecule- and state-specific magnitudes of the transition dipole moments). Additionally, as already commented by others,^29^ the employed laser field should be free of artificial zero-frequency components,^62,63^ which can be achieved in different ways (e.g., deriving the field from the vector potential,^29,64^ or simulating the field explicitly from Maxwell’s equations). We anticipate that our scheme works best for relatively short pulses, for which the approximations (in particular the frozen-nuclei approximation) hold reasonably well, because the “EOE” scheme inherently assumes a spatial locality of the excitation process. However, the simulations for sodium iodide below indicate that pulse durations up to around 100 fs might be described reasonably well for some systems.

#### 2.2.2 Electron-Only Dynamics Simulation

With the vertical excitation data and laser file prepared, we can set up the electron-only dynamics simulations. These simulations are implemented as special SHARC trajectories, propagated using the efficient PySHARC^65^ dynamics driver. The trajectories are initialized in a user-specified state β, usually the ground state, with *c*_α_(0) = δ_βα_. For simplicity, the molecule is defined as a single, stationary dummy atom. Surface hopping and decoherence correction are turned off. The representation of the electronic basis states can in principle be chosen freely here as the electron-only dynamics is purely quantum-mechanical. However, the representation matters for the initial state selection process described below.

This set of simplifications means that the EOE simulations are essentially direct, numerical solutions of the electronic Schrödinger equation for the relevant electronic Hamiltonian (eq 5). The general numerical solution of this equation can be written as

$$|\Psi \left(t\right)\rangle =\mathrm{T̂}e^{-i/\hbar \int_{0}^{t}d\tau \left({Ĥ}_{fz}+{Ĥ}^{L}\left(\tau \right)\right)}|\Psi \left(0\right)\rangle ,$$ 7

in which T̂ is the time-ordering operator, ${Ĥ}_{fz}$ is the electronic Hamiltonian corresponding to the frozen-nuclei approximation (possibly including couplings like spin–orbit couplings), and ${Ĥ}^{L}$ is the time-dependent interaction with the laser pulse. Within SHARC, this time propagation is carried out using a time-ordered product of short-time exponentials of the Hamiltonian.^43^

The electronic structure data for these trajectories are provided by a SHARC interface^66^ that returns identical data in every time step, independent of the passed geometry. The returned data is directly read from the preceding vertical excitation calculation (energies, dipole moments, and optionally spin–orbit couplings). Gradients and nonadiabatic coupling vectors are set to zero and the wave function overlap matrix to a unit matrix. In the absence of any (spin–orbit^35^ or light–matter) couplings, the state populations |*c*_α_(*t*)|^2^ would be constant in these simulations. However, the inclusion of a laser pulse produces time-dependent populations |*c*_α_(*t*)|^2^, according to the states’ transition dipole moments and the relative orientation with respect to the laser pulse. As any kind of electronic coupling can be included here, the “EOE” scheme is very flexible.

Practically, we anticipate that not all matrix elements of the dipole matrices are needed here. First, static dipole moments ${\mathrm{\mu ⃗}}_{\alpha \alpha ,k}$ can in principle lead to the appearance of the dynamic Stark effect, where the oscillating electric field leads to oscillating excitation energies. However, using very weak laser fields, as discussed above, will effectively remove the influence of the dynamic Stark effect. Hence, the static dipole moments can potentially be neglected during the electron-only simulations. This is, e.g., relevant if the electronic structure data is computed with time-dependent density functional theory (TD-DFT) or other response methods, in which static excited-state dipole moments add considerable computational cost. Second, also excited-state–excited-state transition dipole moments ${\mathrm{\mu ⃗}}_{\alpha >0,\beta >0}$ are anticipated to play only a negligible role in most cases, because the induced excited-state absorption is often nonresonant and scales nonlinearly with intensity,^67^ thus becoming negligible with the weak pulses applied in the “EOE” scheme. Thus, excited-state–excited-state transition dipole moments and the corresponding higher-lying electronic states can be neglected unless anticipated to play a role. Having the freedom to neglect the excited-state–excited state transition dipole moments is advantageous if the electronic structure method cannot provide these quantities (e.g., from some TD-DFT codes).

#### 2.2.3 Initial State Selection Process

The electron-only dynamics simulations are carried out with the goal of obtaining the time evolution of the electronic populations |*c*_α_(*t*)|^2^ for each initial condition *k*. The core step in our new excitation scheme is obtaining the initial electronic state for *k* and the corresponding excitation time *t*′ for the subsequent nonadiabatic trajectories. This process involves two smaller steps, which are the renormalization of the population and the application of the surface hopping algorithm to the populations (as discussed below).

This is the first step in the excitation scheme in which the choice of electronic representation matters. In SHARC,^43^ one commonly has two possible choices here. The first choice is the representation of the electronic states as obtained from the electronic structure, i.e., which are spin-free and not field-dressed (this basis is typically called the MCH representation within SHARC^43^). The second choice is the representation of the eigenstates of the full electronic Hamiltonian (called the diagonal representation^43^), which are spin-mixed if spin–orbit couplings are included and field-dressed if light–matter interactions are present. Here, it is important to realize that the subsequent TSH simulations will be performed without including the laser field. Hence, the diagonal representation is not identical in the electron-only simulations and the TSH simulations, such that the selected initial state would be inconsistent. Therefore, the initial state selection described in the following is carried out in the MCH representation (see also ref 35 for further discussions). We note that the choice to select the initial state in the MCH representation is the standard approach in SHARC, even if the actual TSH simulations are carried out in the diagonal representation. Here, during the first time step of the TSH simulation, the active diagonal state is chosen as the one that most strongly overlaps with the selected MCH state. The initial coefficients of the selected MCH state are set to 1 and then transformed into the diagonal representation.^68–70^ In this way it can be assured that at *t* = 0 the initial electronic wave function has a well-defined spin, which is important for simulating intersystem crossing.

To find the largest acceptable renormalization constant for the electronic populations, we analyze the likelihood of exciting from the state β (the state in which the electron-only dynamics is initialized) to all other states (which are the potential initial states for the subsequent TSH simulation). The probability to leave β at time step *t* is given by

8 $$P_{\beta \to any}\left(t\right)=\max\left(0,1-\frac{{\left|c_{\beta }\left(t+\Delta t\right)\right|}^{2}}{{\left|c_{\beta }\left(t\right)\right|}^{2}}\right).$$

From this, one can find the total probability to leave state β in any time step of the simulation as the complementary probability of remaining in β for all steps

9 $$P_{tot,k}=1-\prod_{t}\left(1-P_{\beta \to any,k}\left(t\right)\right)=1-\prod_{t}\min\left(1,\frac{{\left|c_{\beta ,k}\left(t+\Delta t\right)\right|}^{2}}{{\left|c_{\beta ,k}\left(t\right)\right|}^{2}}\right).$$

We note that this *P*_tot,*k*_ is equal to the sum of the population of the excited states after the pulse ended, but assuming that no population was transferred back to β (this is the effect of the min function).

In order to have the maximum acceptance ratio in the excitation sampling, this probability should be as high as possible. However, there will be large differences in *P*_tot,*k*_ across the different initial conditions *k*—for some *k*, the excitation energies/transition dipole moments will be in resonance/parallel with the laser field, favoring population transfer to the excited states. Meanwhile, for other *k* nearly no population transfer will take place. Thus, the optimal acceptance ratio will be reached if we take the global maximum of *P*_tot,*k*_ over all *k*

$$P_{\max}=\underset{k}{\max}{\;P}_{tot,k}.$$ 10

This value quantifies the probability to get excited to any excited state for the initial condition *k* where this is most likely. This value *P*_max_ is the sought-after renormalization constant.

In the next step, we apply the renormalization constant to the time-dependent excited-state populations for all initial conditions *k* and all excited states α

$${\left|{\mathrm{c̃}}_{k,\alpha }\left(t\right)\right|}^{2}=\frac{{\left|c_{k,\alpha }\left(t\right)\right|}^{2}}{P_{\max}},\forall \alpha \neq \beta .$$ 11

and rescale the population of β accordingly

$${\left|{\mathrm{c̃}}_{k,\beta }\left(t\right)\right|}^{2}=1-\underset{\alpha \neq \beta }{\sum }{\left|{\mathrm{c̃}}_{k,\alpha }\left(t\right)\right|}^{2}=\frac{|c_{k,\beta }\left(t\right){|}^{2}-\left(1-P_{\max}\right)}{P_{\max}}.$$ 12

Since *P*_max_ will typically be a positive value close to zero, this renormalization significantly increases the population transfer to the excited states. We note that it implies for the differences between two time steps

$${\left|{\mathrm{c̃}}_{k,\alpha }\left(t+\Delta t\right)\right|}^{2}-{\left|{\mathrm{c̃}}_{k,\alpha }\left(t\right)\right|}^{2}=\frac{1}{P_{\max}}\left({\left|c_{k,\alpha }\left(t+\Delta t\right)\right|}^{2}-{\left|c_{k,\alpha }\left(t\right)\right|}^{2}\right)$$ 13

for all α, even including α = β. Thus, we enhance population transfer between the states without otherwise changing the electronic dynamics. Alternatively, the renormalization procedure can be thought of as neglecting the nonexcited initial state population and normalizing the remainder for producing the hopping probabilities.

In the next steps, we compute surface hopping probabilities from the renormalized populations $|{\mathrm{c̃}}_{k,\alpha }\left(t\right){|}^{2}$ in order to stochastically find the initial excited state for the subsequent TSH simulations. Here, we use the GFSH formalism^46^ because it depends only on the populations but not on any other quantity that might not be available during postprocessing. We note that GFSH is independent of the phase of the coefficients, so we can apply the renormalization directly to the populations. Plugging the renormalized populations from eqs 11 and 12 into the GFSH probabilities in eq 3, we obtain

14 $${\mathrm{P̃}}_{\beta \to \alpha }\left(t\right)=\left(1-\frac{{\left|c_{\beta }\left(t+\Delta t\right)\right|}^{2}-\left(1-P_{\max}\right)}{{\left|c_{\beta }\left(t\right)\right|}^{2}-\left(1-P_{\max}\right)}\right)\times \frac{{\left|c_{\alpha }\left(t+\Delta t\right)\right|}^{2}-{\left|c_{\alpha }\left(t\right)\right|}^{2}}{\sum_{\gamma }\;\max\left[0,{\left|c_{\gamma }\left(t+\Delta t\right)\right|}^{2}-{\left|c_{\gamma }\left(t\right)\right|}^{2}\right]}$$

for hopping probabilities from the initial state β. Note that only the prefactor in parentheses is affected by the renormalization, whereas the second factor stays the same as in eq 3. For hopping probabilities from an excited state, ${\mathrm{P̃}}_{\alpha \to \beta }$ or ${\mathrm{P̃}}_{\alpha \to \gamma }$ , the renormalization preserves the unmodified eq 3, because the $\frac{1}{P_{\max}}$ factors in eqs 11 and 13 cancel out in both factors of eq 14.

It can be shown by induction that 1 – *P*_max_ ≤ |*c*_β_(*t*)|^2^ for all time steps and for all trajectories (see Section S2 in the Supporting Information). Consequently, the numerator and denominator of the first factor in eq 14 cannot become negative, such that the renormalization as described does not affect the sign of the hopping probabilities. If the hopping probabilities from eq 3 at a time step *t* are positive, they will also be positive when evaluated with eq 14. Furthermore, evaluating $\frac{{\mathrm{P̃}}_{\beta \to \alpha }\left(t\right)}{P_{\beta \to \alpha }\left(t\right)}$ from eqs 3 and 14 leads to a factor of $\frac{{\left|c_{\beta }\left(t\right)\right|}^{2}}{{\left|c_{\beta }\left(t\right)\right|}^{2}-\left(1-P_{\max}\right)}$ , which for ${\left|c_{\beta }\left(t\right)\right|}^{2}\approx 1$ (as is generally obtained with weak lasers) simplifies to $\frac{1}{P_{\max}}$ . This is the factor by which the renormalization enhances the large majority of hopping probabilities out of the initial (lower) state β of all initial conditions. The hopping probabilities are enhanced by a larger factor if ${\left|c_{\beta }\left(t\right)\right|}^{2}$ approaches 1 – *P*_max_. This makes sense if one considers that ${\left|c_{\beta }\left(t\right)\right|}^{2}\to 1-P_{\max}$ implies ${\left|{\mathrm{c̃}}_{\beta }\left(t\right)\right|}^{2}\to 0$ , and that means that the hopping probabilities need to approach 100% to ensure the highest possible acceptance ratio of initial conditions.

As a side remark, we note that the presented approach to derive enhanced hopping probabilities from the renormalized populations is preferable to a simple uniform rescaling of the original hopping probabilities. This is because a rescaling of the per-time-step hopping probabilities with a constant factor does not produce a uniform rescaling of the total excitation probabilities of each initial condition, as shown in Section S3.

Once the hopping probabilities are computed via eq 14, we can obtain the initial electronic state α_*k*_(0) and starting time *t*_*k*_^′^ for each initial condition *k*. Here, for each *k*, we simply apply the stochastic sampling from eq 4, starting at *t* = 0 and running over all time steps. If a hop is performed to a state α, the hopping probabilities for the remaining steps are recomputed for leaving α, so that hops back to the initial state and hops to other coupled states are possible. The state to which the last hop is performed is taken as α_*k*_(0) and the corresponding time as *t*_*k*_^′^. If the last hop is to the initial state β, the initial condition is not selected, and no trajectory will be started from it. Similarly, initial conditions for which no hops are performed at all are also not considered further.

We note that the inclusion of more than one hop per initial condition in the algorithm is based on practical considerations. Preliminary tests that were restricted to a maximum of one hop per initial condition showed that there is a significant number of off-resonance states that get selected unexpectedly. These states get temporarily populated by the interaction with the laser, but due to being off resonance, they are depopulated in the second half of the pulse, so that no net population remains. Admitting a second hop in the EOE selection algorithm ensures that these off-resonance states are removed from the set of selected initial states. Third hops as well as hops from the excited state to other excited states were exceedingly rare in the examples shown below. Such hops would otherwise introduce some inaccuracies in the description of the excitation, because the nuclei actually move between the different hops, which is not described by the frozen-nuclei EOE simulations.

Once each initial condition *k* has been processed and the initial electronic state α_*k*_(0) and starting time *t*_*k*_^′^ are obtained for each (or determined that *k* is not selected at all), this information is written to an output file, using the standard file format for SHARC initial conditions. As part of this work, the file format was slightly extended to allow storing the starting time *t*_*k*_^′^. Here we note that we consider each initial condition *k* only once, ensures that each excited initial condition is fully statistically independent.

#### 2.2.4 Full Dynamics Simulation

After the initial state selection process, the excitation-scheme-specific steps are finished. Subsequently, the actual TSH trajectories (with moving nuclei) are set up normally, using α_*k*_(0) as the initial electronic state. The initial electronic coefficients are set as $c_{\alpha ,k}\left(0\right)=\delta_{\alpha \alpha_{k}\left(0\right)}$ in the MCH representation, as mentioned above. As the effect of the excitation laser has already been incorporated by means of the initial state, the TSH trajectories do not need to include the laser explicitly in the Hamiltonian.

During the setup, the time of excitation *t*_*k*_^′^ is written into a small file in each trajectory folder. Once all TSH trajectories are finished, the trajectories can be shifted in time according to *t*_*k*_^′^ during postprocessing. This allows to recover the true temporal distribution of the excitation process for finite laser pulses. This time shifting aspect of our proposed excitation scheme is essentially the same as in ref 29.

## 3 Computational Details

To showcase the proposed “EOE” excitation scheme, we carried out several sets of simulations on the sodium iodide (NaI) system using the SHARC package^8,37^ including the newly implemented “EOE” scheme. The nonadiabatic dynamics of NaI is well-known historically for its role in the development of ultrafast pump–probe spectroscopy^71^ and quantum dynamics simulations.^72–74^ Moreover, it has been used in the previous decades as an important test system for various nonadiabatic molecular dynamics methods.^29,75–77^ Here, we employ it due to its useful combination of nontrivial excited-state dynamics, the availability of analytical models for the potential energy surfaces,^72^ low dimensionality that makes exact quantum dynamics calculations feasible, and its use in previous studies investigating excitation schemes.^29,77^

In brief (details below), based on a single set of initial geometries/velocities, we generate several different sets of initial conditions with differently obtained initial states based on different laser pulses. We use three excitation energies (low: 3.68 eV, mid: 3.89 eV, high: 4.15 eV) and three pulse durations (intensity FWHM’s of 20 fs, 100 fs, and 500 fs), equivalent to the test simulations for the “PDA” scheme in ref 29. For comparison purposes, we generate three additional sets of initial conditions with the “vertical” excitation scheme (low/mid/high excitation energy ±0.03 eV). From these 12 sets of initial conditions, we launched TSH simulations to obtain the time-dependent mean ${\left\langle R\right\rangle }_{S_{1}}$ and standard deviation ${\left\langle \Delta R\right\rangle }_{S_{1}}$ of the Na–I distances. Reference results were obtained with nine grid-based quantum dynamics simulations (low/mid/high excitation energy, each with 20 fs/10 fs/500 fs pulses).

### 3.1 Potential Energy Surfaces and Quantum Dynamics Simulations

We used a diabatic two-state Hamiltonian reported in the 1970s and 1980s for NaI.^78,79^ The corresponding equations are given in Section S4. The quantum dynamics simulations used a spatial grid of 1024 points between 3 and 40 Bohr. The ground state vibrational wave function was obtained via imaginary time propagation and served as initial wave function for the QD simulations. The dynamics simulations were carried out with the split-operator propagator, using a 0.1 fs time step. Convergence was checked against a calculation with 4096 grid points and 0.01 fs time steps; deviations in ⟨*R*⟩ and ⟨Δ*R*⟩ were <0.01 Å.

Overall, we performed nine quantum dynamics simulations to serve as reference for the TSH simulations.

### 3.2 Laser Pulses

The nine laser pulses were generated based on this analytical functional form (see Section S5 in the Supporting Information^39,63,80^ )­

$$\overset{\to }{E}(t)=E_{0}\cdot e^{-\frac{4\ln2}{{FWHM}^{2}}{\left(t-t_{c}\right)}^{2}}\cdot \cos\left(\omega_{0}\left(t-t_{c}\right)+\theta \right)\mathrm{e⃗}.$$ 15

Here, $E_{0}$ is the maximum electric field amplitude, FWHM is the full-width-at-half-maximum of the electric field amplitude/envelope, *t*_c_ is the temporal center of the envelope, θ is the phase at *t* = *t*_c_, and **e⃗** is the polarization unit vector. The pulses had central energies of 3.68 eV (called “low”), 3.89 eV (“mid”), and 4.15 eV (“high”). The intensity FWHM’s were 20 fs, 100 fs, and 500 fs. The maximum electric field strength was set to 0.001 au and the phase was θ = 0. The center of the pulses was shifted to *t* = 0 in all figures below. The polarization vector was chosen parallel to the NaI molecular axis.

### 3.3 Phase Space Sampling and Spectrum

We sampled 10,000 initial conditions from the numerically computed Wigner distribution^52,54^ of the ground state vibrational wave function (i.e., at 0 K) obtained from imaginary time propagation as mentioned above. All subsequent calculations were based on this one set. For each geometry, a single-point calculation was carried out using the analytical model. From this data, we simulated the absorption spectrum as a Gaussian convolution with full width at half-maximum (FWHM) of 0.1 eV. The spectrum is shown in Figure 2.

**Figure 2:**
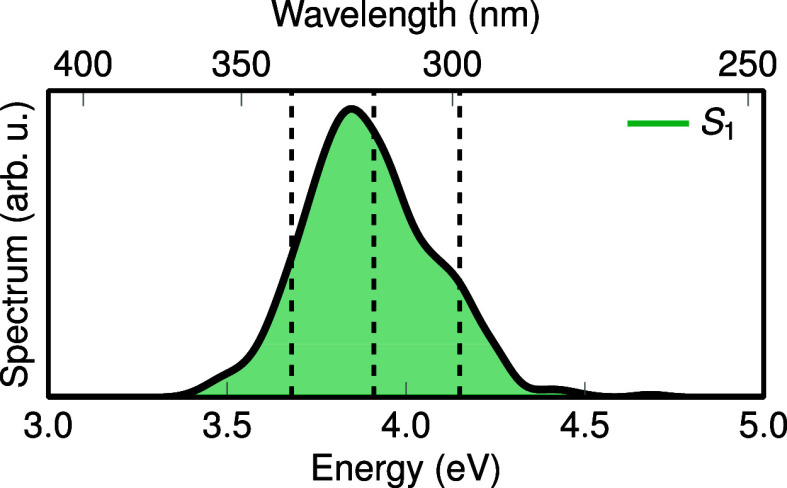
Simulated absorption spectrum of NaI computed from 10,000 Wigner samples using the analytical model. The dashed lines indicate the central energies of the low-, mid-, and high-energy laser pulses.

### 3.4 Initial State Selection

Initial conditions with the “vertical excitation” scheme were selected from three energy windows, 3.65–3.71 eV, 3.86–3.92 eV, and 4.12–4.18 eV, using selection probabilities for each electronic state proportional to $\frac{f_{osc}}{\Delta E^{2}}$ .

For the initial conditions prepared with the “EOE” scheme, each of the nine laser pulses was used. The electron-only simulations were carried out with a 0.0625 fs time step and propagated until the electric field was effectively zero. The laser polarization was not randomized. As described above, in these simulations, nuclear motion was ignored, the same electronic energies and dipole moments reused in all time steps, and hopping and decoherence corrections were turned off. From the results of these trajectories, the initial electronic state and starting time were obtained stochastically (based on eq 14) for all 10,000 initial conditions. We note that only every eighth step of the electron-only simulations was written out, making the selected start times multiples of 0.5 fs, i.e., multiples of the time step of the subsequent TSH simulations. The results of the initial state selection was analyzed by means of scatter plots of the excitation energy and excitation time of the accepted initial states, and a comparison of the scatter plots with the pulses’ Wigner distribution.

### 3.5 Nonadiabatic Dynamics Simulations

Based on the 12 sets of initial conditions (three from “vertical” excitation, nine from “EOE”), we launched TSH simulations, including two states. The nuclear time step was 0.5 fs, and the electronic wave function was propagated with 0.02 fs steps using local diabatization, using wave function overlaps computed from the adiabatic-diabatic transformation matrices at subsequent time steps. The TSH trajectories were propagated for between 1600 and 2200 fs, depending on the length of the laser pulses. No explicit laser was included here.

The simulations used SHARC surface hopping probabilities^43^ (rather than GFSH probabilities that are used within the “EOE” scheme), an energy-based decoherence correction^81^ with the recommended decoherence parameter value of 0.1 a.u., and rescaling of the full velocity vectors along their direction.^8,47,82,83^ Trajectories were analyzed by computing the time-dependent average and standard deviation of the Na–I distance for each swarm.

## 4 Results and Discussion

In the following, we discuss the selection of the initial electronic states in the employed excitation schemes, the obtained nonadiabatic dynamics of NaI, as well as the agreement with the quantum dynamics reference calculations and the “PDA” excitation scheme.^29^

### 4.1 Excited-State Selection

We first discuss the energetic and temporal distribution of the selected initial conditions from the “EOE” scheme for the low-energy, 20 fs pulse. Here, we compare our distribution to one obtained with the “PDA” scheme, because the “vertical” scheme cannot be used to compare starting times.

The comparison between “EOE” and “PDA” is given in Figure 3. In panel a, we show the simulated absorption spectrum of NaI, the intensity spectrum of the employed pulse, and histograms of the distribution of excitation energies of selected initial states. We can see that “EOE” and “PDA” give consistent results. Both select initial states only from within the laser’s energetic spectrum, as expected. The most likely excitation energy agrees approximately with the central energy of the laser. Furthermore, the histograms show a stronger tail at high energies, consistent with the absorption spectrum of NaI that shows higher intensity at energies above the laser central energy. Given that we used a constant diabatic transition dipole moment, this higher spectral intensity arises from a higher occurrence of large excitation energies in the Wigner distribution of the ground state.

**Figure 3:**
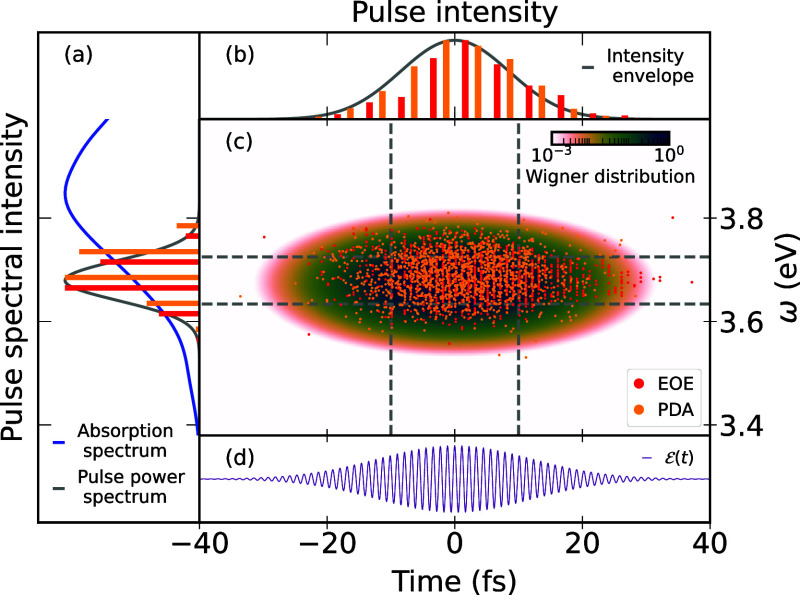
Comparison of the distribution of excitation energies and excitation times for the low-energy, 20 fs laser pulse between the “EOE” and “PDA”^29^ schemes. Panel (a) shows the absorption spectrum of NaI (blue, from Figure 2), the pulse spectra intensity (gray), and histograms of the excitation energies of the selected initial states. Panel (b) shows the temporal intensity envelope of the pulse and histograms of the excitation times of the selected initial states. Panel (c) shows scatter plots of the excitation energies and times for the two methods, together with the Wigner distribution of the laser pulse (note the logarithmic color scale). Panel (d) shows the pulse’s electric field over time for reference.

In panel b, we show the temporal envelope of the pulse together with histograms of the excitation time. Both excitation schemes produce similar results, although the excitation probability before the pulse center is somewhat reduced for the “EOE” scheme. The scatter plots in panel c likewise indicate a similar performance of the “EOE” and “PDA” schemes; however, the former scheme produces a triangle-shaped tail for on-resonance transitions. We discuss these observed differences in more detail below. We note that in the “EOE” scheme, we only pick excitation times that are a multiple of the time step of the subsequent TSH simulations, to aid in the statistical analysis of the time-shifted trajectories. This is the reason that the “EOE” results in panel c exhibit a discrete distribution of starting times. Overall, both excitation schemes produce consistent excitation distributions, indicating that the “EOE” scheme correctly describes the excitation process and the nuclear density of the excited/promoted wave function.

In Figure 4, we present additionally all excitation energy–excitation time scatter plots for all nine laser pulses, each computed with the “EOE” scheme. The labels in the corner of each panel indicate the number of initial conditions that were selected for each laser pulse. For reference, using the “vertical” excitation scheme, 848, 1356, and 474 initial conditions were selected for the low-, mid-, and high-energy selection windows (with windows of ±0.03 eV width, i.e., similar to the 20 fs pulse). It can be noted that the EOE scheme with the 20 fs pulse produces the largest number of selected initial states, compared to the 100 and 500 fs pulses. This can be explained by the spectral bandwidth of the different pulses. The number of selected initial states is reduced by approximately a factor of 5 between the 20 and 100 fs pulses (and the 100 and 500 fs pulses), in accordance with the inverse proportionality of spectral bandwidth and temporal width.

**Figure 4:**
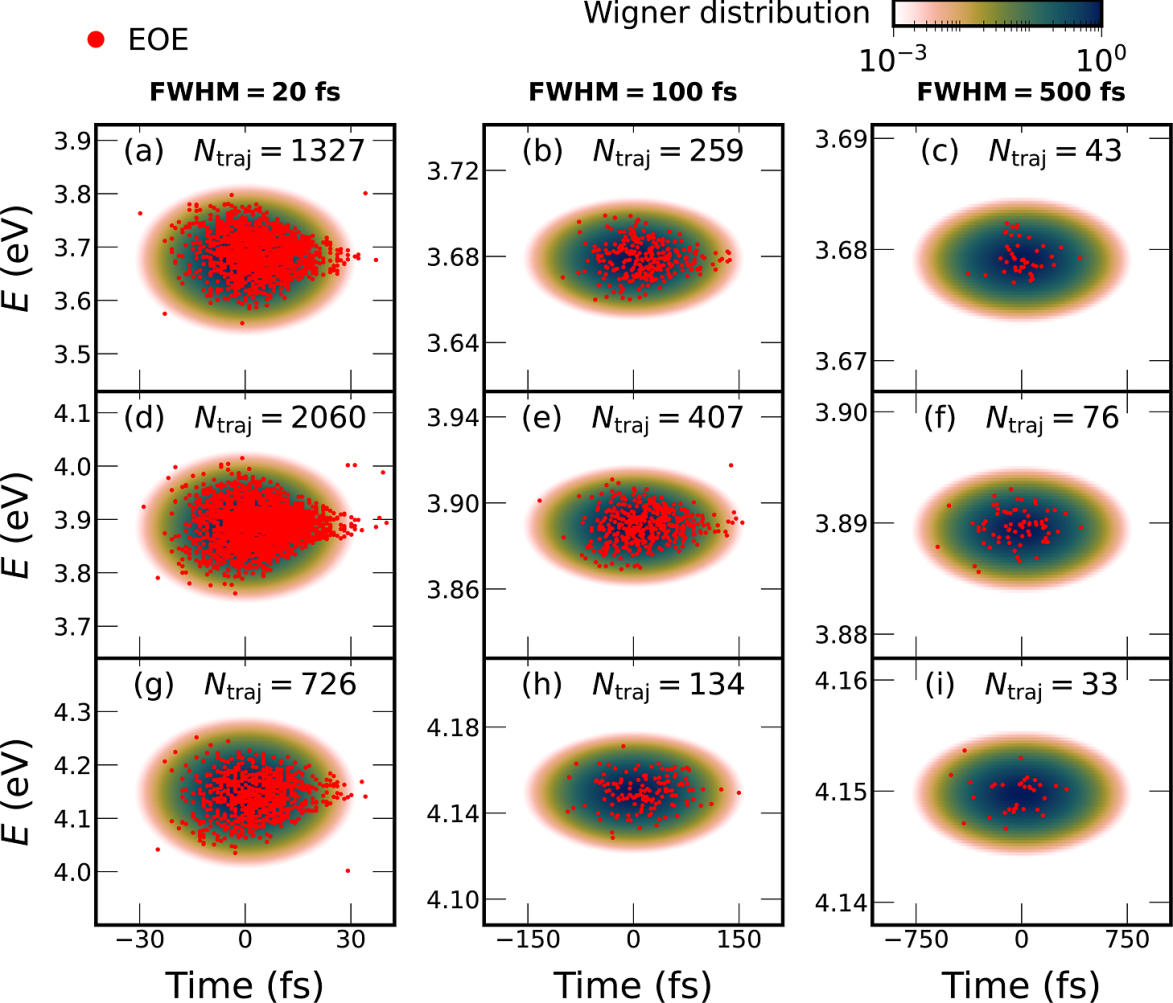
Comparison of the Wigner distributions of the nine used laser pulses with the energetic and temporal distribution of the selected initial conditions from the “EOE” selection scheme. Note that each column of panels uses the same time axis, while the energy axes are different for each panel. The labels give the number of selected initial states, based on a set of 10,000 initial geometries/velocities.

Besides the number of selected initial conditions, Figure 4 shows the distribution of excitation energy and starting time. It can be seen that, in each case, the excited initial states generally fall within the Wigner distribution of the pulse. However, especially for the panels with a large number of selected initial conditions (panels a,d), the scatter plot shows a teardrop-shaped distribution, with a reduced selection probability at early times and a triangular tail at late times. This triangular tail also indicates that, for off-resonance transitions, essentially no starting times near the end of the pulse were obtained.

The teardrop-shaped distribution of selected initial conditions can be explained by an analytical model of the excitation process that takes place within each electron-only simulation. We assume coherent excitation of a static two-state system, using first-order perturbation theory, the rotating wave approximation, a pulse of the form $E\left(t\right)=E_{0}e^{-t^{2}/2\tau^{2}}\;\cos\left(\omega_{0}t\right)$ , and a unit coefficient in the ground state before the pulse. It can be shown that the coefficient of the upper state is proportional to the integral over the pulse envelope, which for a Gaussian pulse relates to the (complex) error function.^84,85^ The electronic coefficient of the upper state is $c_{\alpha }\left(t\right)=-\sqrt{2\pi }i\frac{\mu_{\beta \alpha }E_{0}\tau }{4\hbar }e^{-\tau^{2}\Omega^{2}/2}\left(1+\mathrm{erf}\left(\frac{t}{\sqrt{2}\tau }-\frac{i\tau \Omega }{\sqrt{2}}\right)\right)$ , where Ω = ω_0_ – ω_βα_ is the detuning of the laser from the transition β → α. Computing the time derivative of the population |*c*_α_|^2^ gives

$$\frac{\partial }{\partial t}{\left|c_{\alpha }\right|}^{2}\left(t;\Omega \right)=\sqrt{2\pi }\frac{\mu_{\beta \alpha }^{2}E_{0}^{2}\tau }{4\hbar^{2}}e^{-\frac{t^{2}}{2\tau^{2}}-\frac{\tau^{2}\Omega^{2}}{2}}\times R\left[e^{it\Omega }\left(1+\mathrm{erf}\left(\frac{t}{\sqrt{2}\tau }-\frac{i\tau \Omega }{\sqrt{2}}\right)\right)\right].$$ 16

For the two-state case, |*c*_β_(*t*)|^2^ = 1 – |*c*_α_(*t*)|^2^, hence the derivative in eq 16 is formally proportional to the hopping probabilities in eq 8 (with negative probabilities set to zero).

Figure 5 plots eq 16 together with the marginal probabilities in time and frequency. It can be observed that the shape of the distribution only depends on τ, which simply stretches/compresses it; hence, we plot eq 16 in reduced coordinates $\frac{t}{\tau }$ and τ·Ω. The figure reveals that the analytical model in eq 16 produces a teardrop-shaped distribution of the excitation probability that is fully consistent with the scatter plots in Figures 3 and 4. Minor differences between the model in eq 16 and actual distributions from the “EOE” scheme can possibly arise from the accounting for additional electronic states, second-order coupling between excited states, and geometry-dependent transition moments that are not considered by eq 16. Figure 5 raises several implications for the excitation probability distributions. The most probable excitation time (black dots) depends on the detuning of the respective transition from the central laser frequency: on-resonance transitions tend to get excited slightly after the pulse center. For this reason, the temporal marginal probability (panel a) is slightly shifted to later times compared to the pulse envelope. This is because for on-resonance transitions the population grows with the *square* of the accumulated pulse intensity over time, i.e., follow a |1 + erf­(*t*)|^2^ behavior. In contrast, the frequency marginal probability (panel b) exactly matches the pulse’s power spectrum.

**Figure 5:**
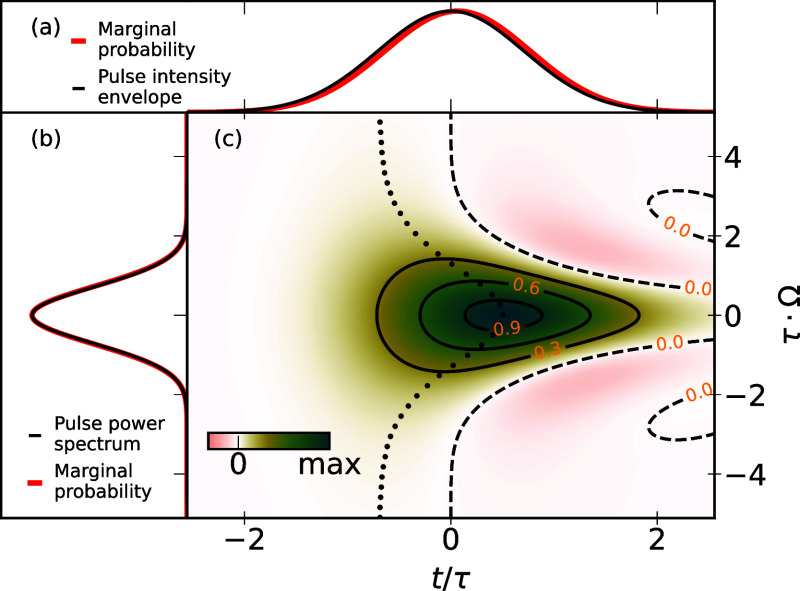
Plot of eq 16, the temporal derivative of the upper state population for the coherent excitation in a two-level system, with reduced time *t*/τ and reduced energy detuning τ·Ω. Panels (a,b) show the time and frequency marginal (i.e., integrated) probabilities, while panel c shows the full distribution. In panel (c) black dots mark the time of maximum excitation probability for each detuning.

The analytical model illustrates that the observed distribution of initial conditions is a consequence of treating the excitation process fully coherently and with a constant transition energy for each initial condition in the “EOE” scheme. This coherent treatment explains the differences between the excitation distributions in Figure 5 and the pulse Wigner distribution in Figure 3. Whether the assumption of a fully coherent excitations is justified depends on the molecular system under investigation. We expect thus that the “EOE” scheme works best for systems where excitation energies are only weakly geometry-dependent in the Franck–Condon region, like in *f*–*f* transitions in lanthanide complexes.^86^ For systems where the assumption of coherent excitation is not reasonable, we anticipate that adding a decoherence correction to the “EOE” scheme would produce more time-localized excitation rates. In such a scheme, the distribution of excitation time and energy would be more similar to the Wigner distribution of the pulse and, consequently, more comparable to the results of the PDA scheme.

### 4.2 Nonadiabatic Dynamics

The main results of the nonadiabatic dynamics simulations and quantum dynamics simulations are shown in Figure 6, which presents the expectation values (panels a–d) and standard deviations (panels e–h) of the swarm of trajectories (or wave packets). Plots of the time-dependent nuclear densities from the TSH and quantum dynamics simulations are given in Figure S1 in Section S6 of the Supporting Information. In Figure 6, panels a and e show the results for the TSH simulations launched from the “vertical” initial conditions in three different energy windows (the width of the window corresponds to the width of the 20 fs pulse). It can be seen that already this rather crude excitation scheme can correctly describe the evolution of the bond length expectation value. However, as shown in panel e, the swarms of trajectories are significantly too narrow, compared to the quantum dynamics reference (dashed lines).

**Figure 6:**
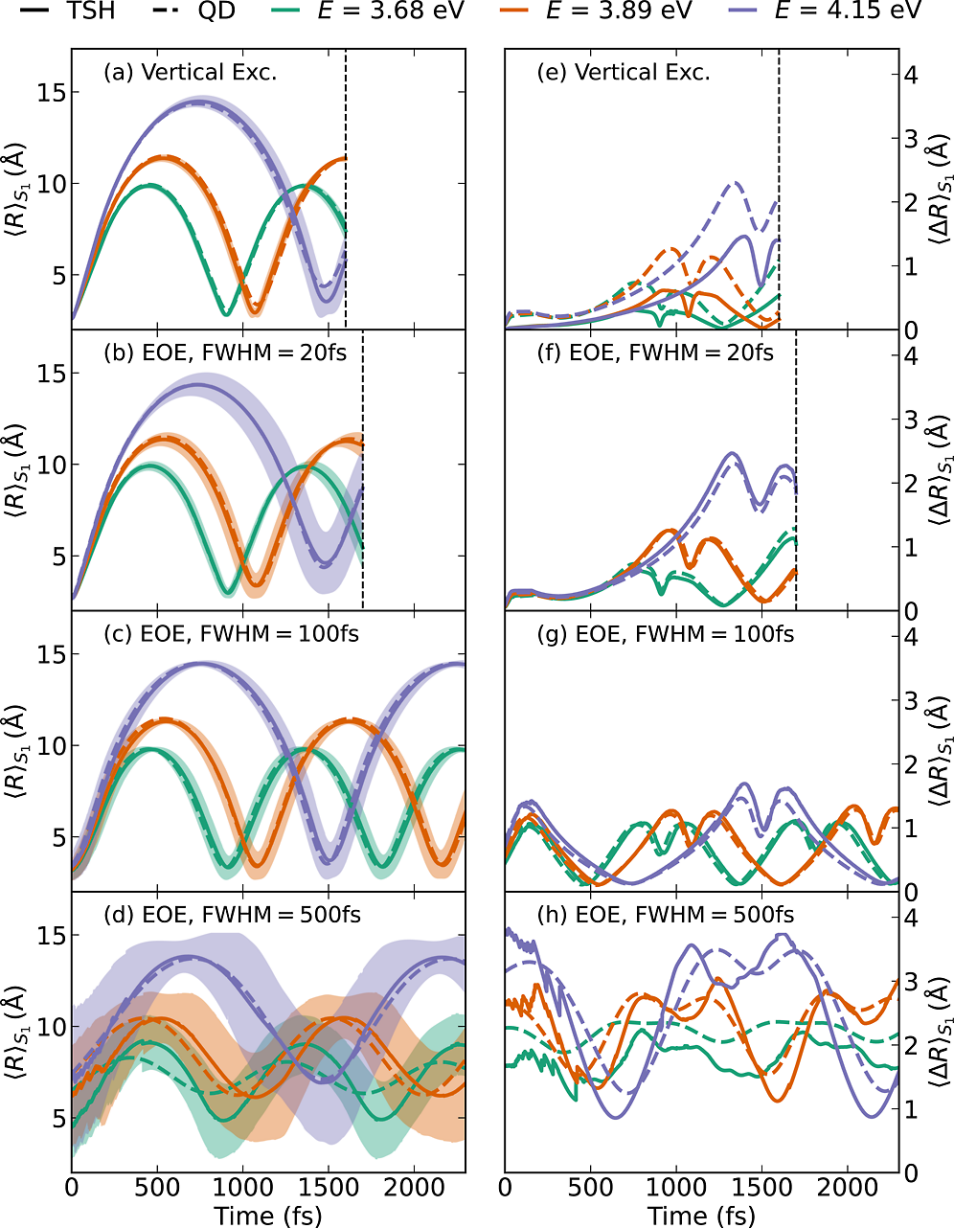
Comparison of the temporal evolution of the expectation values ${\left\langle R\right\rangle }_{S_{1}}$ (a–d) and standard deviation ${\left\langle \Delta R\right\rangle }_{S_{1}}=\sqrt{{\left\langle R^{2}\right\rangle }_{S_{1}}-{\left\langle R\right\rangle }_{S_{1}}^{2}}$ (e–h) of the Na–I bond length. We show results for the “vertical” excitation scheme (a,e) and the “EOE” scheme with 20 fs (b,f), 100 fs (c,g), and 500 fs (d,h) pulses. Dashed lines show the reference quantum dynamics (QD) results, and full lines plus shaded areas represent the TSH results. The dashed lines in a,e were taken from the 20 fs pulse quantum dynamics simulation. Corresponding nuclear density plots are given in Figure S1.

The results obtained with the “EOE” scheme provide generally a better agreement with the reference results. In panels b,f, the results for the 20 fs pulse are shown. While the “EOE” scheme improves the bond length expectation values only moderately over the “vertical” scheme, the improvement for the standard deviations is very notable. Thus, similarly accurate results as with the “PDA” scheme^29^ are obtained for short laser pulses. For the somewhat longer 100 fs pulse, the “EOE” scheme still produces reasonable results (again of similar accuracy as the PDA scheme), as evidenced in panels c and g. Finally, the results for the long 500 fs pulse in panels d and h clearly show the limitations of the excitation scheme, even though some qualitative agreement is still achieved for the high-energy pulse (see Figure S1 for why the agreement is only qualitative). However, we note that sodium iodide is a system with rather heavy atoms and rather flat potential energy surfaces, where the electronic energies are not expected to vary very quickly, so that longer pulses might lead to smaller deviations than in other systems. Further applications of the scheme to other molecular systems, e.g., some of the benchmark systems used in the nonadiabatic dynamics community,^75,87^ will show under which conditions the “EOE” scheme provides the most robust results.

### 4.3 Application to a Molecular System

To show that the “EOE” scheme is also applicable to polyatomic systems, in Section S7 and Figure S2 we present simulations on the CBQ molecule, a rigid analogue of the “molecular Tully II model” 4-(*N*,*N*-dimethylamino)­benzonitrile.^87^ CBQ is described by a 5-state linear vibronic coupling model^88^ with full rotational and translational invariance^89^ at the TDA-ωB97X-D/def2-TZVP level of theory.^87^ 10,000 Wigner samples were generated and excited with either the “vertical excitation” scheme (5.95–6.05 eV window) or the “EOE” scheme with a laser pulse with 6.0 eV central energy and 18.2 fs temporal FWHM (corresponding to a 0.1 eV spectral FWHM). The selected initial conditions were propagated with SHARC for 120 fs.

The results in Figure S2a–c show strong population oscillations (20 fs period) between the adiabatic S_3_ and S_4_, which, however, are mostly washed out when applying the time shifting described in Section 2.2.4. The peak population of the S_3_ is reduced from over 75% to about 50%, highlighting the importance of explicitly including the photoexcitation process when comparing to experiment. Additionally, we show in Figure S2d–f that the “EOE” scheme can be used to prepare initial conditions including a realistic distribution of partially aligned molecules with respect to a linearly polarized pump laser. Such initial conditions can be used to, e.g., simulate polarization-dependent scattering experiments.^90^

## 5 Conclusions

We have presented a scheme to sample the initial electronic state for TSH simulations according to the interaction with an external laser pulse. The scheme is based on electron-only quantum dynamics simulations (i.e., for frozen nuclear coordinates) that explicitly include the relevant light–matter interaction Hamiltonian and can also include other contributions to the electronic Hamiltonian. Once the electron-only dynamics simulations are finished, the evolution of the electronic populations is renormalized (to increase the acceptance ratio) and used to derive surface hopping probabilities, which are used to stochastically identify the initial electronic state and start time for each geometry. The only required input are the sampled initial geometries, excitation energies and transition dipole moments at these geometries, and a laser pulse. This scheme is effectively an approximation to TSH simulations that explicitly include the laser pulse in the propagation, but the new scheme is much more economic and about as affordable as other commonly used excitation schemes.^26,29^ The main advantages of the scheme are (i) that it can include various waveforms, (ii) that arbitrary interactions can be included in the electronic Hamiltonian (e.g., beyond-electric-dipole interactions relevant for nonplane-wave beams or high photon energies, spin–orbit couplings), and (iii) that initial conditions with polarization-induced anisotropy can be created. As it is based on the coherent propagation of the electronic coefficients, it is most appropriate for molecular systems with slow Franck–Condon motion after excitation, but should work generally well for sufficiently short pulses.

We have demonstrated, based on TSH simulations of sodium iodide, that the “EOE” scheme can accurately describe the excitation and impart the effect of specific laser pulses on the nuclear dynamics, with good agreement with quantum dynamics simulations. In a second example on the CBQ molecule, we have shown that the “EOE” scheme offers a way to include temporal broadening in the results of TSH simulations, and that it can be used to prepare initial conditions that are partially aligned to a linearly polarized pump laser. The examples also showed that the proposed excitation scheme is computationally very cheap. The frozen-nuclei SHARC simulations take only a few seconds for each initial conditions, thanks to the efficient PySHARC implementation,^65,66^ and the excited-state selection scheme likewise provides little overhead. The code used in this work is available under the GNU Public License as part of the SHARC 4.0.2 release,^57^ and it will be fully integrated and documented in the upcoming SHARC 4.1 package.

## Supplementary Material



## Notes

Workflow of the “EOE” scheme, proof by induction related to eq 14, example of renormalizing versus rescaling the hopping probabilities, NaI potential energy surfaces, definitions of laser pulses, nuclear density plots for NaI, results on CBQ (PDF)
