# Supplementary material for: An Excitation Strategy for the Initial Condition Generation for Surface Hopping Trajectories Using Electron-Only Dynamics Including Explicit Laser Pulses
Source: J Chem Theory Comput. 2025 Dec 8;21(24):12741–54. doi: 10.1021/acs.jctc.5c01390 (PMC12746453; doi:10.1021/acs.jctc.5c01390)
Supplement: Supplementary file 1 [file ct5c01390_si_001.pdf]

Supporting Information: An excitation strategy for the initial condition generation for surface hopping trajectories using electron-only dynamics including explicit laser pulses

Lorenz Grünewald,[†,‡] Laurens van Dam,[†,‡] and Sebastian Mai[*,†]

†*Institute of Theoretical Chemistry, Faculty of Chemistry, University of Vienna, 1090 Vienna, Austria*

‡*Vienna Doctoral School in Chemistry (DoSChem), Faculty of Chemistry, University of Vienna, 1090 Vienna, Austria*

E-mail: sebastian.mai@univie.ac.at

Contents

S1 Workflow of the "EOE" excitation scheme in SHARC 4.0.1 **S-3**

S2 Proof by Induction for the constant sign of hopping probabilities **S-4**

S3 Renormalization of populations versus rescaling of hopping probabilities **S-6**

S4 NaI potential energy surfaces **S-9**

S5 Definition of the laser pulses **S-10**

S6 Nonadiabatic dynamics: Density plots for NaI **S-11**

S7 Nonadiabatic dynamics: 6-cyanobenzquinuclidine **S-13**

References **S-15**

S1 Workflow of the “EOE” excitation scheme in SHARC 4.0.1

In brief, the following steps need to be carried out in the SHARC 4.0.1 release to prepare and analyze trajectories based on the “EOE” scheme:

1. Generate initial geometries and velocities as usual, e.g., with `amber_to_initconds.py` or `wigner.py`. This produces an `initconds` file with initial conditions $k = 1 \ldots N$.

2. Choose a level of theory, prepare the required template file (and potentially initial orbitals), and use `setup_init.py` to perform single-point calculations for all N initial conditions with a given number of states. This produces a folder for each initial condition k, in which the corresponding SHARC interface needs to be run. This produces a `QM.out` file in each folder.

3. Use `laser.x` to create a `laser` file containing the time-dependent electric field.

4. Use the interactive script `setup_laser_excitation.py` to set up the electron-only dynamics simulations. This script functions similar to the standard setup script (`setup_traj.py`), but sets several options automatically. It sets up N SHARC trajectories using the `SHARC_QMOUT.py` interface for frozen nuclei, which uses the `QM.out` files from step 2. Furthermore, the setup script will utilize the `initconds` and `laser` files. During setup, users should use the MCH representation.

5. Each electron-only dynamics simulation now needs to be executed, using `driver.py -i QMOUT input`. The automatically created run scripts can be used. Note that by default, the electron-only dynamics simulations are run with PySHARC and with NetCDF output for optimal performance. The simulations will produce the standard SHARC output files, including `output.dat`.

6. In the same folder where step 4 was carried out, call `excite_laser_excitation.py`. This will run the data extraction for all electron-only simulations, extract the electronic populations, obtain $P_{\max}$, renormalize the populations of all simulations, and perform the sampling of the initial state and starting time. The results are written into a new initial conditions file, e.g., `initconds_Singlet_1.excited`.

7. Using the `initconds_Singlet_1.excited` file and `setup_traj.py`, trajectories can be set up normally. The only difference is that a `start.time` file is written in each trajectory folder. Trajectory execution is identical to other excitation schemes.

8. During analysis of results, `data_extractor.x` and `data_extractor_NetCDF.x` as well as `geo.py` and `geo_NM.py` automatically recognize the `start.time` files and apply the time shift to all output data. `shift_output_lis.py` can be used to produce a shifted `output.lis` file.

9. The swarm analysis tools `population.py` and `data_collector.py` can be used to compute various ensemble results based on the time-shifted output data.

S2 Proof by Induction for the constant sign of hopping probabilities

Let $P_{\text{tot}}(T)$ be the total surface hopping probability to leave the initial state β between time zero and T (we leave out the index k from the main text for brevity). Similar to Eq. (9) in the main text, the definition is:

$$P_{\text{tot}}(T) = 1 - \prod_{t}^{T} \min\left(1, \frac{p_\beta(t+\Delta t)}{p_\beta(t)}\right), \tag{S1}$$

where we write $p_\beta(t) = |c_\beta(t)|^2$. We want to show by induction that the counter probability, i.e., the probability to remain in the initial state β until T is always equal or less than the population of β at T:

Statement: We want to show that

$$1 - P_{\text{tot}}(T) = \prod_{t}^{T} \min\left(1, \frac{p_\beta(t+\Delta t)}{p_\beta(t)}\right) \leq p_\beta(T+\Delta t) \quad \forall T, \tag{S2}$$

with the initial ground state probability $p_\beta(0) = 1$.

Base case: The base case corresponds to $T = 0$:

$$\prod_{t}^{0} \min\left(1, \frac{p_\beta(t+\Delta t)}{p_\beta(t)}\right) = \min\left(1, \frac{p_\beta(\Delta t)}{p_\beta(0)}\right) \leq p_\beta(\Delta t). \tag{S3}$$

Given that $p_\beta(0) = 1$ and that $p_\beta(\Delta t) \leq 1$, we arrive at:

$$p_\beta(\Delta t) \leq p_\beta(\Delta t), \tag{S4}$$

which is true.

Inductive hypothesis: The inductive hypothesis is

$$\prod_{t}^{T} \min\left(1, \frac{p_\beta(t+\Delta t)}{p_\beta(t)}\right) = \min\left(1, \frac{p_\beta(T+\Delta t)}{p_\beta(T)}\right) \prod_{t}^{T-\Delta t} \min\left(1, \frac{p_\beta(t+\Delta t)}{p_\beta(t)}\right) \leq p_\beta(T+\Delta t), \tag{S5}$$

assuming that

$$\prod_{t}^{T-\Delta t} \min\left(1, \frac{p_\beta(t+\Delta t)}{p_\beta(t)}\right) \leq p_\beta(T) \tag{S6}$$

is true.

Inductive step: We do a case distinction here.

Case 1: The ground state population does not increase from T to $T + \Delta t$:

$$p_\beta(T + \Delta t) \leq p_\beta(T). \tag{S7}$$

In this case, we obtain:

$$\frac{p_\beta(T + \Delta t)}{p_\beta(T)} \prod_{t}^{T-\Delta t} \min\left(1, \frac{p_\beta(t + \Delta t)}{p_\beta(t)}\right) \leq p_\beta(T + \Delta t). \tag{S8}$$

Multiplying both sides by $\frac{p_\beta(t)}{p_\beta(t+\Delta t)}$ directly gives Eq. (S6), making the inductive hypothesis true for Case 1.

Case 2: The ground state population increases from T to $T + \Delta t$:

$$p_\beta(T + \Delta t) > p_\beta(T). \tag{S9}$$

In this case, we obtain:

$$1 \cdot \prod_{t}^{T-\Delta t} \min\left(1, \frac{p_\beta(t + \Delta t)}{p_\beta(t)}\right) \leq p_\beta(T + \Delta t). \tag{S10}$$

Considering Eqs. (S6) and (S9), this is always true:

$$\prod_{t}^{T-\Delta t} \min\left(1, \frac{p_\beta(t + \Delta t)}{p_\beta(t)}\right) \leq p_\beta(T) < p_\beta(T + \Delta t). \tag{S11}$$

Conclusion: This shows that:

$$1 - P_{\text{tot}}(T) = \prod_{t}^{T} \min\left(1, \frac{p_\beta(t + \Delta t)}{p_\beta(t)}\right) \leq p_\beta(T + \Delta t) \quad \forall T. \tag{S12}$$

We also know that:

$$P_{\max} = \max_{k,T} P_{\text{tot},k}(T) \tag{S13}$$

for all times T and all electron-only simulations k. Thus, we can write:

$$1 - P_{\max} \leq P_{\text{tot},k}(T) \quad \forall k, T. \tag{S14}$$

Hence, we can deduce

$$|c_\beta(t)|^2 - (1 - P_{\max}) > 0 \quad \forall t, \tag{S15}$$

which is the statement used in the main text after Eq. (14).

S3 Renormalization of populations versus rescaling of hopping probabilities

As shown in the main text, the renormalization of the electronic populations in Eqs. (11)–(13) effectively rescales the hopping probabilities from the initial state β by $\frac{|c_\beta(t)|^2}{|c_\beta(t)|^2-(1-P_{\max})}$. At first glance, it is unclear why the hopping probabilities cannot simply be rescaled directly, e.g., by a factor of $\frac{1}{P_{\max}}$. Here, we show that these two procedures—renormalization of the populations and recomputation of the hopping probabilities *versus* directly rescaling the hopping probabilities—are not equivalent.

This can be shown by a simple numerical example with two states, for which we repeat here the main working equations. For two states, the original hopping probability from state β based on the original coefficients is given as:

$$P(t+\Delta t) = 1 - \frac{|c_\beta(t+\Delta t)|^2}{|c_\beta(t)|^2}. \tag{S16}$$

The renormalized population of β is:

$$|\tilde{c}_\beta(t)|^2 = \frac{|c_\beta(t)|^2 - (1-P_{\max})}{P_{\max}} \tag{S17}$$

and the corresponding hopping probabilities are:

$$\tilde{P}(t+\Delta t) = 1 - \frac{|c_\beta(t+\Delta t)|^2 - (1-P_{\max})}{|c_\beta(t)|^2 - (1-P_{\max})} = \frac{|c_\beta(t)|^2}{|c_\beta(t)|^2 - (1-P_{\max})} P(t+\Delta t). \tag{S18}$$

The directly rescaled hopping probabilities are given by:

$$\bar{P}(t+\Delta t) = \frac{P(t+\Delta t)}{P_{\max}}. \tag{S19}$$

In the case that the hopping probabilities are directly rescaled, it is now interesting to derive the populations that would lead to the rescaled probabilities. These can be found by inverting Eq. (S16) ($|c_\beta(t+\Delta t)|^2 = [1-P(t+\Delta t)]\cdot|c_\beta(t)|^2$) and assuming $|c_\beta(0)|^2 = 1$ to get:

$$|\bar{c}_\beta(t)|^2 = \prod_{\tau=1}^{t}(1-\bar{P}(\tau)). \tag{S20}$$

(we note that computing the populations from the hopping probabilities is only possible if the population of β is decreasing monotonically, due to setting negative probabilities to zero)

Using these equations, we can construct, for any example time series of original populations $|c_\beta(t)|^2$, the original hopping probabilities $P(t+\Delta t)$, the renormalized hopping probabilities $\tilde{P}(t+\Delta t)$, and the rescaled hopping probabilities $\bar{P}(t+\Delta t)$, as well as the corresponding populations. We show a simple example with three time steps, where the population of β is starting at 1 and is then reduced to 0.75 and then to 0.5. The renormalization and rescaling

Table S1: Comparison of hopping probabilities from renormalization vs. simple probability scaling, assuming $P_{\max} = 0.5$, for a simulation with very high population transfer.

Line	Procedure	$t = 0$	$t = 1$	$t = 2$
Original populations and probabilities:				
(1)	Original $\lvert c_\beta(t)\rvert^2$	1.00	0.75	0.50
(2)	$P(t)$ computed from Line (1) via Eq. (S16)	—	25.0%	33.3%
Renormalize populations:				
(3)	$\lvert\tilde{c}_\beta(t)\rvert^2$ renormalized from Line (1) via Eq. (S17)	1.00	0.50	0.00
(4)	$\tilde{P}(t)$ computed from Line (1) via Eq. (S18)	—	50.0%	100.0%
Directly rescale probabilities:				
(5)	$\bar{P}(t)$ computed from Line (2) via Eq. (S19)	—	50.0%	66.7%
(6)	$\lvert\bar{c}_\beta(t)\rvert^2$ reconstructed from Line (5) via Eq. (S20)	1.00	0.50	0.167

is done with $P_{\max} = 0.5$ to maximize the population transfer.

The example is shown in Table S1. Line (1) shows the original populations and Line (2) the corresponding hopping probabilities to leave β, which are 25% and 33.3%. We note that the total population to stay in β is $(1 - 0.25) \cdot (1 - 0.33) = 0.5$, consistent with the populations at the last time step.

In Line (3) we apply the renormalization procedure from the main text. Essentially, the population of the excited state (not shown) is multiplied by two, and the population of β is reduced correspondingly. The hopping probabilities are in Line (4). Again, by construction the the total population to stay in β, given by $(1 - 0.5) \cdot (1 - 1) = 0$, is consistent with the renormalized population of β.

Line (5) contains the rescaled hopping probabilities, which are simply twice as large as the ones in Line (2). The populations that are consistent with these hopping probabilities are given in Line (6). As one can see, the final population of β is $(1 - 0.5) \cdot (1 - 0.667) = 0.167$. Hence, doubling the hopping probabilities did not result in twice as much total population transfer from β to the excited state.

It can thus be followed that directly rescaling of the hopping probabilities of all EOE simulation results by a constant factor of $\frac{1}{P_{\max}}$ generally does not simply scale the total excitation probability by the same factor. Instead, the total probability for EOE simulations with relatively large population transfer (on the order of $P_{\max}$) would be scaled by a smaller effective factor that for EOE simulations with very little population transfer. This would then lead to an underestimation of the overall selection probabilities for on-resonance transitions and conversely the selection of too many off-resonance transitions. The effect on EOE simulations with low population transfer can be seen in Table S2, where renormalization and rescaling produce much more similar results than in Table S1.

Instead, the renormalization procedure outlined in the main text ensures that the total excitation probabilities of all initial conditions are scaled uniformly.

Table S2: Comparison of hopping probabilities from renormalization vs. simple probability scaling, assuming $P_{\mathbf{max}} = 0.5$, for a simulation with low population transfer

Line	Procedure	$t = 0$	$t = 1$	$t = 2$
Original populations and probabilities:				
(1)	Original $\lvert c_\beta(t)\rvert^2$	1.00	0.95	0.90
(2)	$P(t)$ computed from Line (1) via Eq. (S16)	—	5.0%	5.26%
Renormalize populations:				
(3)	$\lvert \tilde{c}_\beta(t)\rvert^2$ renormalized from Line (1) via Eq. (S17)	1.00	0.90	0.80
(4)	$\tilde{P}(t)$ computed from Line (1) via Eq. (S18)	—	10.0%	11.11%
Directly rescale probabilities:				
(5)	$\bar{P}(t)$ computed from Line (2) via Eq. (S19)	—	10.0%	10.53%
(6)	$\lvert \bar{c}_\beta(t)\rvert^2$ reconstructed from Line (5) via Eq. (S20)	1.000	0.900	0.805

S4 NaI potential energy surfaces

The diabatic Hamiltonian is defined as

$$\mathbf{H}_{\text{dia}}(R) = \begin{pmatrix} V_{11}(R) & V_{12}(R) \\ V_{12}(R) & V_{22}(R) \end{pmatrix}, \tag{S21}$$

where the adiabatic potential energy surfaces (PESs) are obtained as the eigenvalues of $\mathbf{H}_{\text{dia}}(R)$. R is the Na–I distance. The analytical forms of the diabatic matrix elements are:[S1,S2]

$$V_{11}(R) = \left(A_2 + \left(\frac{B_2}{R}\right)^8\right) e^{-R/r_0} - \frac{e^2}{R} - \frac{e^2(\lambda_+ + \lambda_-)}{2R^4} - \frac{C_2}{R^6} - \frac{2e^2\lambda_+\lambda_-}{R^7} + D_e, \tag{S22}$$

$$V_{12}(R) = A_{12}\,\exp\left[-\,b_{12}\,(R - R_x)^2\right], \tag{S23}$$

$$V_{22}(R) = A_1\,\exp[-\,b_1\,(R - R_0)]\,. \tag{S24}$$

The diabatic transition dipole moment is defined as a constant off-diagonal element directed along the internuclear axis,

$$\boldsymbol{\mu}_{\text{dia}} = \begin{pmatrix} \mathbf{0} & \mu_0\,\hat{\mathbf{R}} \\ \mu_0\,\hat{\mathbf{R}} & \mathbf{0} \end{pmatrix}. \tag{S25}$$

Table S3 lists all parameter values.

Table S3: Parameters used in Eqs. (S22) **to** (S25)**.**

Symbol	Value	Unit
A_2	2760.0	eV
B_2	2.398	eV$^{1/8}$·Å
C_2	11.3	eV·Å^6
λ_+	0.408	Å^3
λ_-	6.431	Å^3
r_0	0.3489	Å
D_e	2.075	eV
e^2	14.3996	eV·Å
A_1	0.813	eV
b_1	4.08	Å^{-1}
R_0	2.67	Å
A_{12}	0.055	eV
b_{12}	0.6931	Å^{-2}
R_x	6.93	Å
μ_0	0.1	a.u.

S5 Definition of the laser pulses

The laser pulses employed in this work were generated with a dedicated tool that is part of the SHARC 4 package. Here we briefly summarize the equations that define the laser pulse. As this work is restricted to the dipole approximation (although the excitation scheme generally can work beyond that approximation), we only define the local electric field without any positional dependence.

The local electric field $\mathcal{E}(t)$ is given as the product of an envelope function $G(t)$, a carrier function $f(t)$, and a normalized polarization vector $\vec{\mathbf{e}}$:

$$\vec{\mathcal{E}}(t) = \Re \left(G(t) f(t) \right) \vec{\mathbf{e}}. \tag{S26}$$

For an unchirped pulse, the envelope is defined as:

$$G(t) = \mathcal{E}_0 \mathrm{e}^{-\frac{(t-t_c)^2}{2\tau^2}} \tag{S27}$$

where $\mathcal{E}_0$ is the peak field strength, t_c is the temporal center, and the parameter τ is

$$\tau = 2\sqrt{2 \ln 2}\, \mathrm{FWHM} \tag{S28}$$

Note that FWHM/$\sqrt{2}$ is thus the FWHM of the intensity envelope.[S2,S3] The carrier is defined as:

$$f(t) = \mathrm{e}^{\mathrm{i}(\omega_0 (t-t_c)+\theta)}, \tag{S29}$$

where ω_0 is the central angular frequency and θ is the carrier-envelope shift (which is generally regarded as zero in this work).

The instantaneous spectrum of a laser pulse is nicely visualized by means of the Wigner distribution function $\mathcal{W}(t, \omega)$,[S3–S5] which is proportional to:

$$\mathcal{W}(t, \omega) = \frac{1}{2\pi} \int_{-\infty}^{\infty} \mathcal{E}^* \left(t - \frac{s}{2} \right) \mathcal{E} \left(t + \frac{s}{2} \right) \mathrm{e}^{\mathrm{i}\omega s} \mathrm{d}s. \tag{S30}$$

From the Wigner distribution, one can directly obtain the temporal intensity envelope:

$$I(t) \propto \int_{-\infty}^{\infty} \mathcal{W}(t, \omega) \mathrm{d}\omega \tag{S31}$$

and the power spectrum:

$$\sigma(\omega) \propto \int_{-\infty}^{\infty} \mathcal{W}(t, \omega) \mathrm{d}t. \tag{S32}$$

S6 Nonadiabatic dynamics: Density plots for NaI

Figure S1 shows the time evolution of the nuclear density along the Na–I bond distance coordinate for the different laser pulses employed in the main text. For each of the nine panels a–i, the top half shows the density from using the "EOE" scheme followed by TSH (the number of trajectories for each is the one given in Figure 4. The bottom half of every panel is the quantum dynamics result. The figure shows that the nuclear dynamics is mostly classical for the 20 fs and 100 fs pulses, and that agreement between TSH and quantum dynamics is very good accordingly. For the 500 fs pulse, nuclear motion is clearly dominated by interference and the population of only few vibrational levels, as evidenced by the quantum dynamics results. Consequently, the TSH simulations cannot properly describe this dynamics.

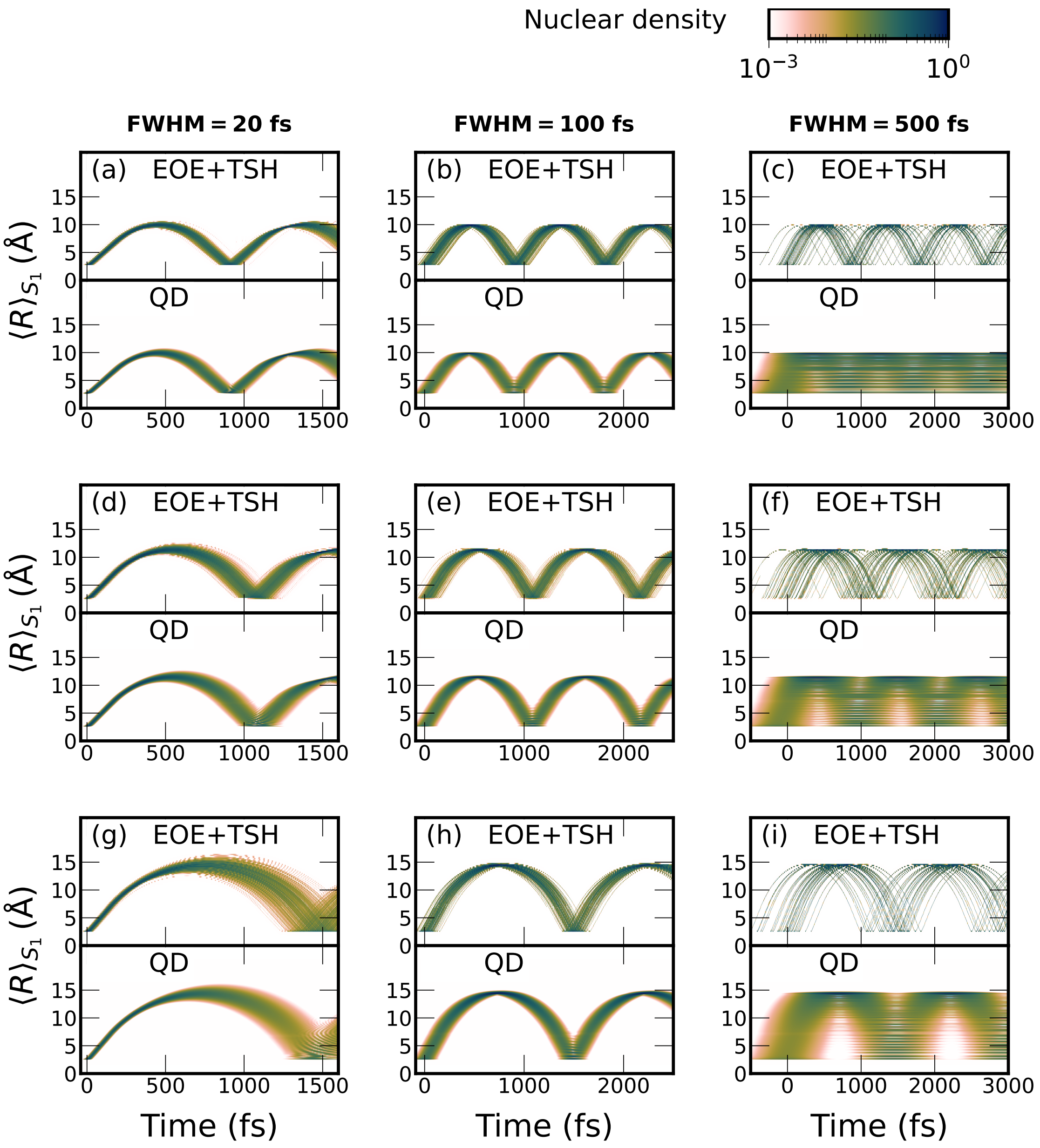

Figure S1: Distribution of the Na–I bond distances in the S_1 state over time after excitation with different laser pulses for the "EOE"+TSH dynamics and quantum dynamics (QD). In the first row (panels a–c), second row (panels d–f), and third row (g–i), the NaI dynamics initiated by laser pulses with central energies of 3.68 eV ("low"), 3.89 eV ("mid"), and 4.15 eV ("high") are shown. In the first column (panels a, d, g)), second column (panels b, e, h), and third column ((panels c, f, i), the laser pulses have a temporal intensity FWHM of 20 fs, 100 fs, and 500 fs, respectively.

S7 Nonadiabatic dynamics: 6-cyanobenzquinuclidine

In this section, we present a second application of the "EOE" scheme. Here, we want to showcase the scheme for a polyatomic molecule. We use the 6-cyanobenzquinuclidine (CBQ) molecule (inset in Fig. S2a), which is a conformationally locked derivative of the widely known 4-(N,N-dimethyl-amino)benzonitrile (DMABN) molecule, which was suggested as a "molecular Tully model".[S6] Both molecules exhibit coherent oscillatory population transfer between two adiabatic states after photoexcitation.

CBQ is a harmonically rigid molecule that can be adequately described by a linear vibronic coupling model, as implemented in the SHARC package.[S7] We have parametrized such a model using the TDA-ωB97X-D/def2-TZVP level of theory,[S6] for five singlet states (S_0 to S_4). From the ground state harmonic oscillator (at the same level of theory), we sampled 10,000 initial conditions. We excited the initial conditions with the "vertical excitation" scheme in the 5.95–6.05 eV energy window, centered on the maximum of the absorption band of the bright $\pi\pi^*$ state (S_3 or S_4 depending on geometry). Additionally, we excited the same initial conditions using the "EOE" scheme, with a laser pulse with a temporal FWHM of 18.2 fs (intensity FWHM of 12.9 fs), central energy of 6 eV, and a maximum field strength of 0.01 au. The simulations were done with randomized polarizations (but for the analysis below we instead rotated the molecule such that the laser is polarized along x). The selected initial conditions ("vertical excitation": 4 in S_2, 395 in S_3, 735 in S_4; "EOE": 4 in S_2, 250 in S_3, 408 in S_4) were used to run SHARC trajectories with the vibronic coupling model, for 120 fs.

Figure S2a–c shows the electronic population of CBQ. Panel a shows the results of the "vertical excitation" scheme. In panel b, we show the results of the "EOE" scheme, but we do not apply the start time shifts to compare with panel a. In panel c, the start time shifts are applied. Panels a and b show consistently strong oscillations (period around 20 fs) between the adiabatic S_4 and S_3 (and S_2 to an extent) in the first 100 fs. As can be seen in panel c, applying a laser pulse with a width of 18 fs washes out most of the oscillations, leading to a maximum S_3 population of 50% (rather than 75%). This shows that it is clearly useful to include an explicit laser pulse in the simulations when the goal is to explain a given experiment.

Figure S2d–f shows another useful feature of the "EOE" scheme when studying excitation by a polarized laser. Panels d–f show the distribution of angles between the molecule's axis (taken as the N–N vector and essentially identical to the direction of the large transition dipole moments) and the coordinate axes, where the laser is x-polarized in the used coordinate system. Since CBQ only exhibits one bright state in our model, all selected initial conditions should have the respective transition dipole moment aligned with the laser polarization. In this case, one expects a $\cos(\theta)^2 \sin(\theta)$ distribution of angle θ between the transition dipole moment and the laser polarization, and a $\sin(\phi)^3$ distribution of angle ϕ between the transition dipole moment and the two directions orthogonal to the laser polarization. These ideal distributions are shown as black curves in Figure S2d–f. As can be seen, the initial conditions selected by the "EOE" scheme follow these distributions consistently. Hence, the "EOE" scheme allows setting up initial conditions that incorporate the partial alignment that is present in the ensemble of excited molecules of a linearly polarized pump laser, which can be used to, e.g., simulate the scattering of such partially aligned ensembles.[S8]

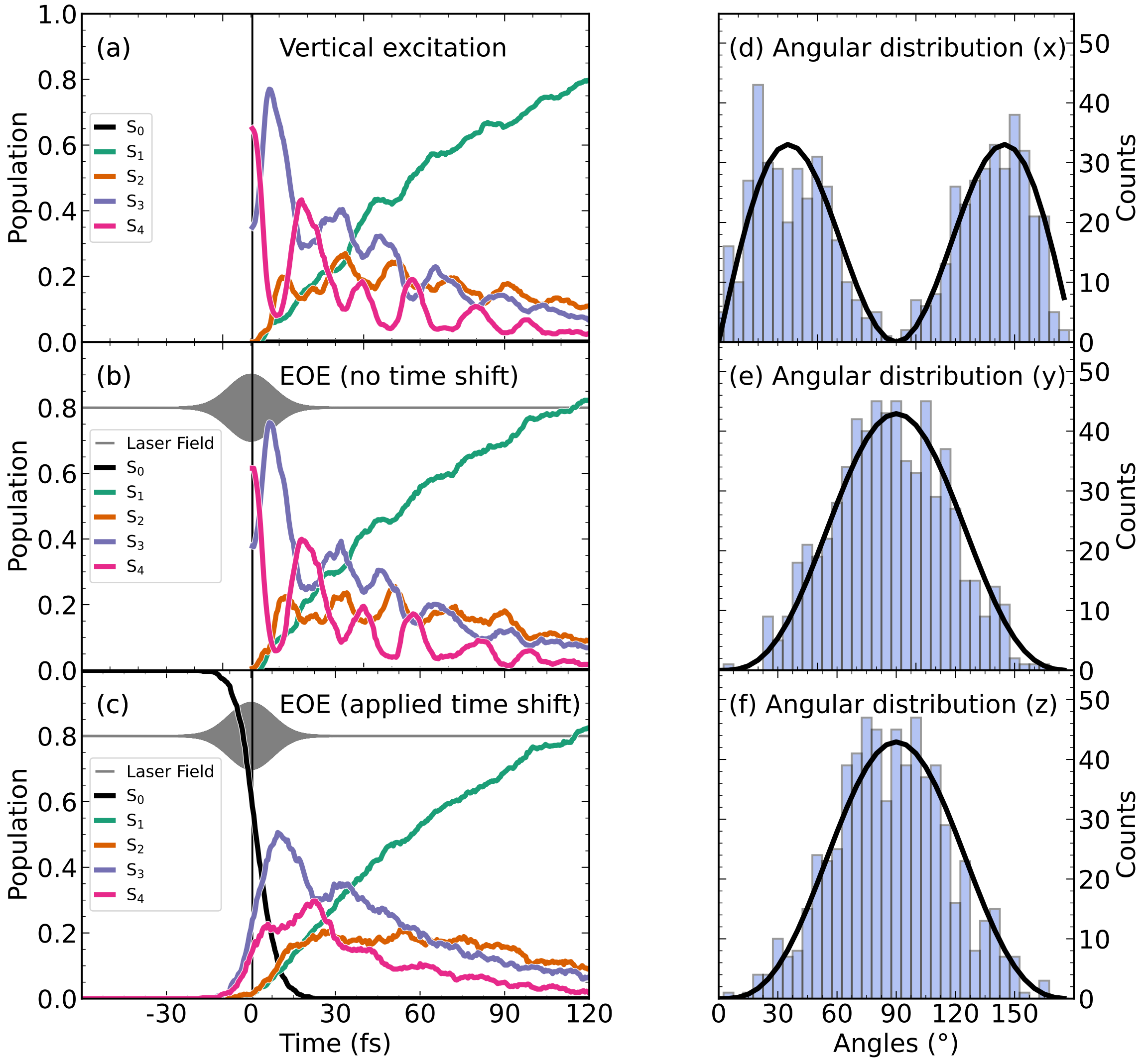

Figure S2: Temporal evolution of the electronic populations of CBQ in the first column: (a) "Vertical excitation" scheme, (b) "EOE" scheme without shifting the trajectories according to the starting times from the electron-only dynamics, (c) "EOE" scheme with trajectories accordingly shifted in time. Distributions of the angle between CBQ's N–N vector and the $x/y/z$ (d/e/f) coordinate axes for initial conditions selected by the "EOE" scheme with an x-polarized laser. The black lines show the idealized expected distribution.

References

(S1) Engel, V.; Metiu, H. A Quantum Mechanical Study of Predissociation Dynamics of NaI Excited by a Femtosecond Laser Pulse. *J. Chem. Phys.* **1989**, *90*, 6116–6128.

(S2) Janoš, J.; Slavíček, P.; Curchod, B. F. E. Including Photoexcitation Explicitly in Trajectory-Based Nonadiabatic Dynamics at No Cost. *J. Phys. Chem. Lett.* **2024**, *15*, 10614–10622.

(S3) Saleh, B. E. A.; Teich, M. C. *Fundamentals of Photonics*, third edition ed.; Wiley Series in Pure and Applied Optics; Wiley: Hoboken, NJ, 2019.

(S4) Siegman, A. E. *Lasers*; University Science Books: Mill Valley, California, 1986.

(S5) Diels, J.-C., Rudolph, W., Eds. *Ultrashort Laser Pulse Phenomena: Fundamentals, Techniques, and Applications on a Femtosecond Time Scale*, 2nd ed.; Optics and Photonics; Elsevier/Academic Press: Amsterdam London, 2006.

(S6) Gómez, S.; Spinlove, E.; Worth, G. Benchmarking Non-Adiabatic Quantum Dynamics Using the Molecular Tully Models. *Phys. Chem. Chem. Phys.* **2024**, *26*, 1829–1844.

(S7) Farkhutdinova, D.; Polonius, S.; Karrer, P.; Mai, S.; González, L. Parametrization of Linear Vibronic Coupling Models for Degenerate Electronic States. *J. Phys. Chem. A* **2025**, *129*, 2655–2666.

(S8) Baskin, J. S.; Zewail, A. H. Oriented Ensembles in Ultrafast Electron Diffraction. *ChemPhysChem* **2006**, *7*, 1562–1574.
